# Identification and Characterization of Cinnamyl Alcohol Dehydrogenase Encoding Genes Involved in Lignin Biosynthesis and Resistance to *Verticillium dahliae* in Upland Cotton (*Gossypium hirsutum* L.)

**DOI:** 10.3389/fpls.2022.840397

**Published:** 2022-04-28

**Authors:** Haipeng Li, Shulin Zhang, Yunlei Zhao, Xulong Zhao, Wenfei Xie, Yutao Guo, Yujie Wang, Kun Li, Jinggong Guo, Qian-Hao Zhu, Xuebin Zhang, Kun-Peng Jia, Yuchen Miao

**Affiliations:** ^1^State Key Laboratory of Cotton Biology, Henan Joint International Laboratory for Crop Multi-Omics Research, School of Life Sciences, Henan University, Kaifeng, China; ^2^College of Biology and Food Engineering, Innovation and Practice Base for Postdoctors, Anyang Institute of Technology, Anyang, China; ^3^State Key Laboratory of Cotton Biology, Institute of Cotton Research, Chinese Academy of Agricultural Sciences, Anyang, China; ^4^Zhengzhou Research Base, State Key Laboratory of Cotton Biology, Zhengzhou University, Zhengzhou, China; ^5^CSIRO Agriculture and Food, Canberra, ACT, Australia

**Keywords:** lignin biosynthesis, CAD, enzymatic assay, monolignols, *Gossypium*, *Verticillium* wilt

## Abstract

Verticillium wilt, caused by the soil-borne fungus *Verticillium dahliae*, is one of the most devastating diseases in cotton (*Gossypium* spp.). Lignin in the cell wall forms a physical barrier to inhibit pathogen invasion, and defense-induced lignification reinforces secondary cell wall to prevent pathogens from further spreading. Cinnamyl alcohol dehydrogenases (CADs) catalyze the production of three main monolignols, *p*-coumaryl- (H), coniferyl- (G), and sinapyl-alcohols (S), which are the fundamental blocks of lignin. Here, we identified *CAD* genes in *G. hirsutum*, analyzed their expression profiles in cotton leaf, stem, and root from different developmental stages, and selected *GhCAD35*, *GhCAD45*, and *GhCAD43*, which were consistently induced by *V. dahliae* inoculation in *G. hirsutum* cultivars resistant or susceptible to *V. dahliae*. On the basis of confirmation of the *in vitro* enzymatic activity of the three proteins in generation of the three monolignols, we used virus-induced gene silencing (VIGS) to investigate the effects of silencing of *GhCAD35*, *GhCAD45*, or *GhCAD43* on resistance to *V. dahliae* as well as on deposition and the composition of lignin. Silencing each of the three *CAD*s impaired the defense-induced lignification and salicylic acid biosynthesis in stem, and compromised resistance to *V. dahliae*. Moreover, our study showed that silencing the three *GhCAD*s severely affected the biosynthesis of S-lignin, leading to a decrease of the syringyl/guaiacyl (S/G) ratio. Heterogeneous overexpression of *GhCAD35*, *GhCAD45*, or *GhCAD43* in *Arabidopsis* enhanced disease resistance. Taken together, our study demonstrates a role of the three *GhCAD*s in defense-induced lignin biosynthesis and resistance to *V. dahliae* in *G. hirsutum*.

## Introduction

Cotton (*Gossypium* spp.) is the most important fiber crop worldwide. Upland cotton (*G. hirsutum* L.) and Sea *Island cotton* (*G. barbadense* L.) are the two domesticated allotetraploid species used for fiber production (Wendel, 1989). Upland cotton constitutes approximately 90% of world’s cotton production. However, most Upland cotton cultivars are susceptible to *Verticillium dahliae*, a soil-borne fungus pathogen causing the disease of *Verticillium* wilt that leads to plant vascular disease and significant economic loss worldwide. *V. dahliae* infects over 200 dicotyledon plant species such as cotton, tobacco, tomatoes, Arabidopsis (Song et al., 2020). Among tomato diseases, *Verticillium wilt* is a major biotic stressor of tomato production (Acharya et al., 2020). In cotton, the average yield loss caused by *Verticillium wilt* is approximately 10–35% annually; therefore it is urgent to understand the interaction mechanism between plants, especially crops, and *V. dahliae*. Most crops infected by *V. dahliae* show typical disease symptoms of leaf chlorosis, wilting, and defoliation, as well as vascular browning and even final plant death (Sink and Grey, 1999; Song et al., 2020). Because the dormant microsclerotia of *V. dahliae* can survive in soil for many years, it is very difficult to control *Verticillium* wilt in field once it occurred (Klosterman et al., 2009). Therefore, breeding of disease-resistant cultivars is considered to be one of the most effective and efficient approaches to control the threat of the pathogen (Cianchetta and Davis, 2015; Sanogo and Zhang, 2016).

Plants possess complex defense systems to counteract pathogens. When perceiving pathogen signals, plants will timely react and transduce the signal through a serial of signaling systems, which eventually lead to the induction of the expression of plant defense related genes and the production of phytoalexin, such as terpenoids and phenylpropanoids (Cui et al., 2000; Tan et al., 2000; McFadden et al., 2001). Phenylpropanoids could act as preformed and inducible antimicrobial compounds during plant defense against pathogens (Dixon et al., 2002; Koeduka et al., 2014; Tohge et al., 2017). The phenylpropanoid pathway synthesizes monolignols, the fundamental blocks of lignin biosynthesis, and phenolic compounds, which are essential for plant disease resistance (Mottiar et al., 2016; Dong and Lin, 2021). Lignin forms a physical barrier by enhancing plant mechanical strength and thickening cell walls to inhibit pathogen invasion and colonization (Xie et al., 2018; Cesarino, 2019); moreover, defense-induced lignification is important for plant innate immunity, which reinforces the secondary cell wall and contributes to pathogen- and insect-resistance (Bhuiyan et al., 2009; Eynck et al., 2012; Chezem et al., 2017). *V. dahliae* induced lignification has been reported to confer resistance to the pathogen in many plant species. For instance, a tomato line LA3038 (*Ve*/*Ve*) resistant to *V. dahliae* exhibited induction of lignin synthesis in *V. dahliae* inoculated roots (Gayoso et al., 2010). Application of elicitor from *V. dahliae* increases the synthesis and deposition of lignin and lignin-like phenolic polymers in cotton hypocotyl tissues (Smit and Dubery, 1997). Transcriptomic study in cotton has also found *V. dahliae* induced up-regulation of lignin biosynthesis genes (Xu et al., 2011). Most recently, *GhMYB4* and *Gh4CL30* were reported to regulate *Verticillium* wilt resistance in cotton by interfering lignification (Xiao et al., 2021; Xiong et al., 2021). Salicylic acid (SA) and jasmonic acid (JA) are necessary for the activation and mediation of plant defense signaling and responses to various biotic stresses (Loake and Grant, 2007; Miao et al., 2019; Zheng et al., 2019; Song et al., 2020). Several studies have shown that lignin associated resistance to *Verticillium* wilt is related to the SA mediated plant defense responses by activation of reactive oxygen species (ROS) signaling (Li et al., 2019; Tang et al., 2019).

Lignin consists of a polymeric structure which forms from the oxidative coupling of different monolignols. *p*- Coumaryl-, coniferyl-, and sinapyl-alcohols are the most prevalent monolignols, giving rise to hydroxyphenyl (H), guaiacyl (G), and syringyl (S) units, respectively, in lignin polymer (Boudet et al., 1995). The relative proportion of these three units in lignin composition is usually up to the plant species, tissue types, and developmental stages (Campbell and Sederoff, 1996). The biosynthesis pathway of monolignols in plants has been well characterized (Dong and Lin, 2021). Cinnamyl alcohol dehydrogenase (CAD) is a class of NADPH-dependent enzymes involved in the last and key step of monolignol biosynthesis (Kim et al., 2004, 2007; Dong and Lin, 2021). Elevated expression of *CAD* gene(s) leads to increased lignin deposition. *CAD* typically presents as a multiple-gene family in plant species. Numerous *CAD* homologues have been described in different plant species with distinct or redundant functions in lignin biosynthesis. The *Arabidopsis* genome contains nine *CADs*, and several of them have been shown to be involved in lignin biosynthesis (Kim et al., 2004, 2007). *AtCAD4* and *AtCAD5* function predominantly and redundantly in lignin synthesis in floral stems, while *AtCAD1* seems only having a minor effect on the lignin content in elongating stems (Sibout et al., 2005). Of the 15 *CADs* identified in *Populus*, *PoptrCAD4* and *PoptrCAD10* are preferentially expressed in xylem, the major tissue of lignin deposition (Barakat et al., 2010; Shi et al., 2010). Cotton *GhCAD7* has a major role in biosynthesis of S-lignin and consequently changes the ratio of syringyl/guaiacyl (S/G) lignin (Zhang S. et al., 2020). Several studies in different species have reported roles of *CADs* in regulation of stress- and pathogen-induced lignification. For instance, three melon *CADs*, i.e., *CmCAD1*, *CmCAD2*, and *CmCAD3*, were found to be induced by drought stress to enhance lignin biosynthesis (Jin et al., 2014; Liu et al., 2020). In *Arabidopsis* and wheat, suppression of the expression of *CAD*s enhances susceptibility to pathogens (Tronchet et al., 2010; Rong et al., 2016). Several *GhCADs* have been shown to be induced upon *V. dahliae* infection (Xu et al., 2011); however, we still know little of the cotton *CAD* genes, their function in lignin biosynthesis, and *Verticillium* wilt resistance.

In this study, we carried out a genome-wide identification of *CAD* genes in *G. hirsutum* and investigated their expression levels in different developmental stage of cotton plants. Three *GhCADs* that are consistently induced by *V. dahliae* infection in *G. hirsutum* cultivars resistant or susceptible to *V. dahliae* were selected for functional characterization. We showed that, based on *in vitro* enzymatic assays, all the three GhCADs possess catalyzing activity. Knocking down the expression level by virus-induced gene silencing (VIGS) compromised *V. dahliae* induced lignin biosynthesis and consequently resistance to *V. dahliae* infection. Therefore, our study demonstrates a clear role of three *GhCAD*s in defense-induced lignin biosynthesis and resistance to *V. dahliae* infection in *G. hirsutum*.

## Results

### Genome-Wide Identification of *CAD* Genes in *G. hirsutum*

**FIGURE 1:**
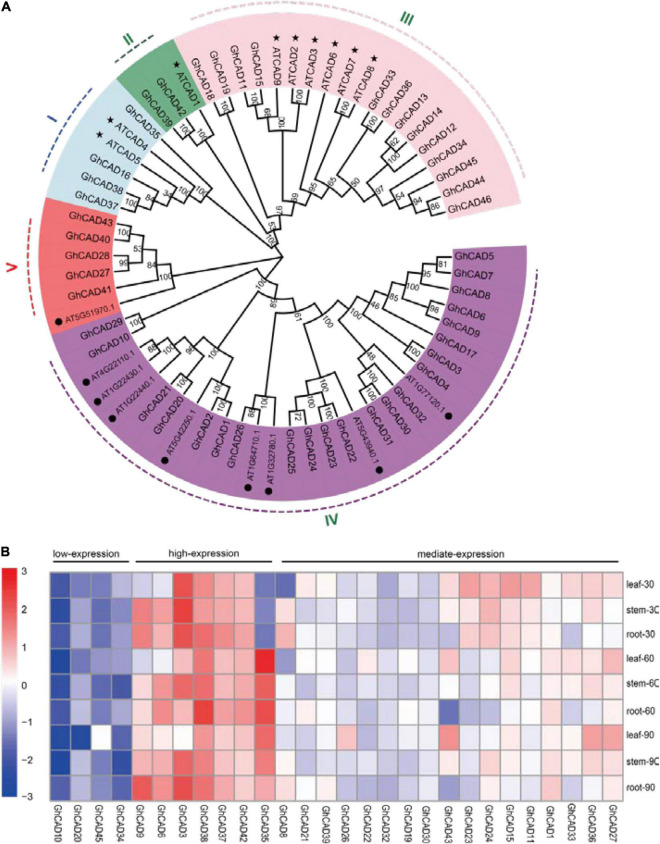
The phylogenetic analysis and tempo-spatial expression pattern of the *CAD* genes in *G. hirsutum*. **(A)** Phylogenetic tree of CAD proteins from Arabidopsis and *G. hirsutum*. The phylogenetic tree was generated by the Neighbor-Joining (NJ) method with 1,000 bootstrap replicates. CAD proteins were divided into I-V groups distinguished by different colors. The nine bona fide CADs of Arabidopsis were marked by “⬤”; the nine candidate CADs of Arabidopsis identified in this study were marked by “★.” **(B)** The tempo-spatial expression pattern of 28 *GhCAD* genes. The expression of different *GhCADs* in the leaf, stem, and root tissues of 30-, 60-, 90-day old plants, respectively. Three biological replicates were performed for each treatment. The expression of *GhCAD*s was relative to that of *GhUBQ7*.

Using the nine *Arabidopsis* CAD protein sequences as queries, we searched the TAIR database^1^ and identified another nine CAD-like proteins in *Arabidopsis* based on domain analysis (Figure 1A). The nine CAD-like proteins were originally annotated as aldehyde dehydrogenases. We then used these *Arabidopsis* CAD and CAD-like protein sequences to search against the proteins annotated in the Upland cotton genome [*Gossypium hirsutum* (AD1) “TM-1” genome NAU-NBI_v1.1_a1.1] (Zhang et al., 2015). All hits were further validated based on the presence of the two key domains (PF08240, alcohol dehydrogenase GroES-like domain; PF00107, zinc-binding dehydrogenase) responsible for the CAD catalytic activity. Finally, we identified 46 cotton CAD and CAD-like proteins which were classified into five groups (group I to V) according to the phylogenetic tree and the conserved motifs analysis of the amino acids of this gene family (Figure 1A and Supplementary Figure 1). Groups I, II, and III contain the nine *Arabidopsis* CADs previously reported and 19 GhCADs. Groups IV and V contain the nine *Arabidopsis* CAD-like proteins identified in this study and 27 GhCAD-like proteins (Figure 1A).

We investigated the tempo-spatial expression pattern of the 46 candidate *GhCADs* in root, stem, and leaf of 30-, 60-, and 90-day old cotton plants (cv. TM-1) by qRT-PCR. Of the 46 genes, 28 were found to be expressed in at least one of the samples tested (Figure 1B). Generally, the 28 *GhCADs* could be classified as low- (4), mediate- (17), and high-expression (7) three groups based on their overall expression levels in the tissues analyzed (Figure 1B). A specific expression pattern was observed for some *GhCADs*, for example, *GhCAD27* and *GhCAD43* seemed to be leaf specific, and *GhCAD6* seemed to be mainly expressed in stem and root, while *GhCAD35* showed an obvious developmental stage specific expression pattern (Figure 1B).

### Identification of *GhCADs* Responding to *V. dahliae* Infection

**FIGURE 2:**
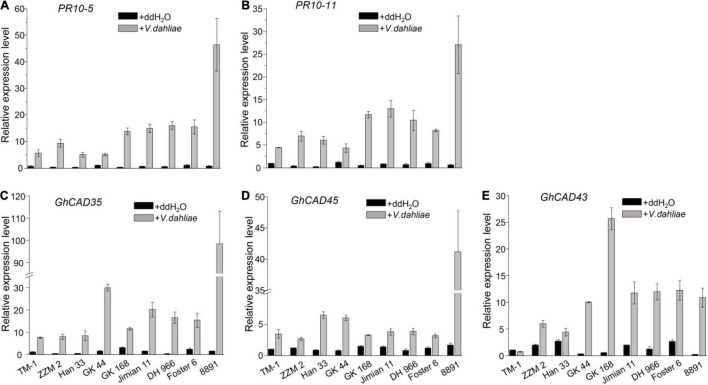
*V. dahliae* inoculation induces the expression of several *GhCADs* in different *G. hirsutum* cultivars. **(A,B)** The transcriptional levels of two pathogen defense-related genes *PR10-5* and *PR10-11* under control (ddH_2_O) and *V. dahliae* treatment conditions in different *G. hirsutum* cultivars. **(C–E)** The transcriptional levels of *GhCAD35*, *GhCAD45*, *GhCAD43*, and *GhCAD40* under control (ddH_2_O) and *V. dahliae* treatment conditions in different *G. hirsutum* cultivars. Four *Verticillium* wilt resistant cultivars (ZZM 2, Han 333, GK 44, and GK168), four *Verticillium* wilt susceptive cultivars (Jimian 11, DH 966, Foster 6, and 8891) and TM-1 were used in the *V. dahliae* inoculation experiment; two-weeks-old plants 18 days after *V. dahliae* inoculation were used for qRT-PCR analysis. Three biological replicates were performed for each treatment. The gene expression level of each gene in TM-1 under the control (ddH_2_O) treatment was normalized as 1. *GhUBQ7* was used as reference gene.

Lignin biosynthesis and deposition in plants induced by *V. dahliae* infection contribute to resistance to the pathogen (Zhang et al., 2019). To identify *GhCADs* responsible for *V. dahliae* induced lignin biosynthesis, we firstly identified four resistant cultivars, including Zhongzhimian 2 (ZZM 2), Han 333, GK 44, and GK 164, and four susceptible cultivars, including Jimian 11, 8891, Foster 6, and DH 966 from a collection of *G. hirsutum* cultivars (Supplementary Figure 2). ZZM 2 and Jiman11 have been previously reported to be resistant and susceptible to *V. dahliae*, respectively (Zhang et al., 2014; Zhang X.Y. et al., 2020). To exaggerate the expression of *GhCADs* after the induction of *V. dahliae* infection, we next compared the expression of the 28 *GhCADs* upon *V. dahliae* infection in the four resistant and the four susceptible cultivars, as well as TM-1, using plants at 18 days-post-infection (dpi) by qRT-PCR. *PR10-5* and *PR10-11* (Dowd et al., 2004), two pathogen defense-related genes, were used as positive controls. As expected, the expression level of *PR10-5* and *PR10-11* was highly up-regulated in all tested cultivars infected by *V. dahliae* (Figures 2A,B). Of the 28 *GhCADs*, three, including *GhCAD35*, *GhCAD45*, and *GhCAD43*, were consistently highly induced in almost all tested cultivars (Figures 2C–E), while the other *GhCADs* were either repressed or no consistent responsive trend in different cultivars after *V. dahliae* inoculation (Supplementary Figures 3, 4).

**FIGURE 3:**
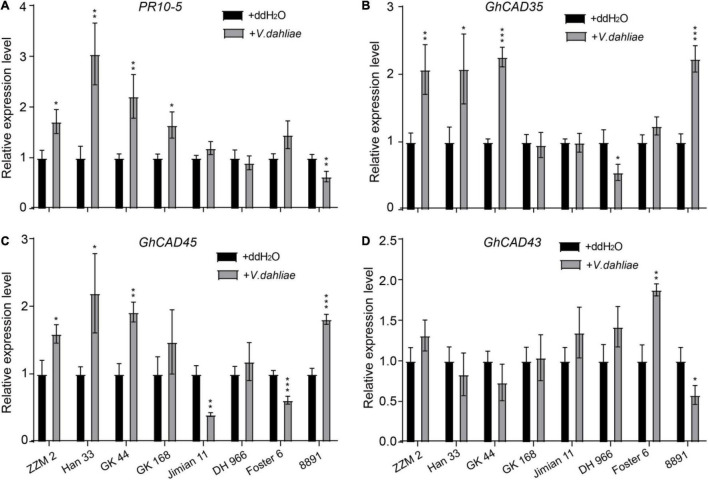
Different expression patterns of *GhCAD35* and *GhCAD45* in *G. hirsutum* resistant- and susceptible-cultivars 24 h after *V. dahliae* inoculation. **(A–D)** The transcriptional levels of *PR10-5*, *GhCAD35*, *GhCAD45*, and *GhCAD43* under the control (ddH_2_O) and *V. dahliae* treatment conditions in different *G. hirsutum* cultivars. Four *Verticillium* wilt resistant cultivars (ZZM 2, Han 333, GK 44, and GK 168) and four *Verticillium* wilt susceptive cultivars (Jimian 11, DH 966, Foster 6, and 8891) were used in the *V. dahliae* inoculation experiment; two-weeks-old plants 24 h after *V. dahliae* inoculation were used in qRT-PCR analysis. Three biological replicates were performed for each treatment. The gene expression level in each cultivar under the control (ddH_2_O) conditions was normalized as 1. *GhUBQ7* was used as reference gene. The data represent the mean ± SD (*n* = 3 biological replicates), *P < 0.05, **P < 0.01, ***P < 0.001 (Student’s *t*-test).

We further investigated the expression profiles of *GhCAD35*, *GhCAD45*, and *GhCAD43*, as well as *PR10-5*, 24 h after *V. dahliae* infection in the four resistant and the four susceptible cultivars. Interestingly, *PR10-5* was significantly induced in all the four resistant but not in the four susceptible cultivars (Figure 3A). *GhCAD35* and *GhCAD45* were significantly induced in three (ZZM 2, Han 33, and GK 44) of the four resistant cultivars and one (8891) of the susceptible cultivars although they were repressed or not affected in different susceptible cultivars (Figures 3B,C); while the expression of *GhCAD43* was only significantly induced in one (Foster6 24) of the cultivars (Figure 3D). Together, these results suggest a potential role of these three genes, particularly *GhCAD35* and *GhCAD45*, in response to *V. dahliae* infection at the early infection stage.

### GhCAD35, GhCAD45, and GhCAD43 Possess Cinnamyl Alcohol Dehydrogenase Activities

**FIGURE 4:**
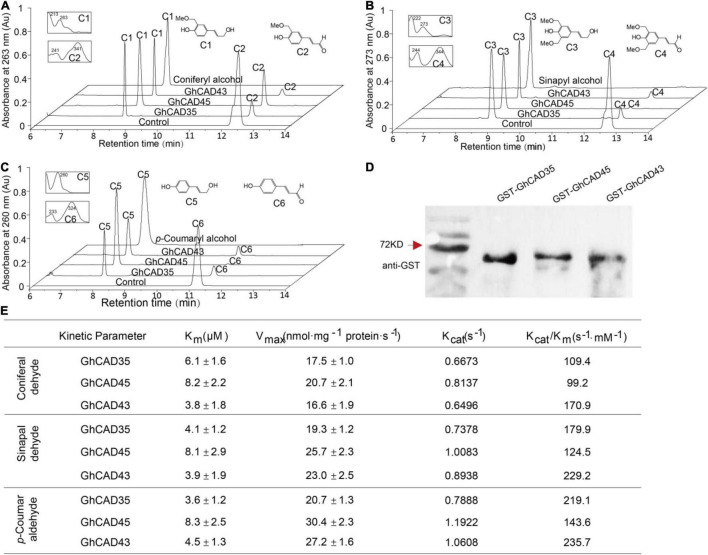
Characterization of the cinnamyl alcohol dehydrogenase (CAD) catalytic activities of GhCAD35, GhCAD45, and GhCAD43. **(A–C)** HPLC analyses of *in vitro* incubation of GhCAD35, GhCAD45, and GhCAD43 with coniferaldehyde, sinapaldehyde and *p*-coumaraldehyde, to yield corresponding monolignols, coniferyl alcohol, sinapyl alcohol, and *p*-coumaryl alcohol, respectively. C1-C6 indicate coniferyl alcohol, coniferaldehyde, sinapyl alcohol, sinapaldehyde, *p*-coumaryl alcohol, and *p*-coumaraldehyde, respectively. Controls in A-C were incubated with GST protein. **(D)** Western blot showing the levels of recombinant GhCAD35, GhCAD45, or GhCAD43 protein fused with a GST tag. GST antibody was used. **(E)** The catalytic parameters of GhCAD35, GhCAD45, and GhCAD43 toward the substrates *p*-coumaraldehyde, coniferaldehyde, and sinapaldehyde, respectively.

In plants, CADs catalyze the conversion of *p*-coumaraldehyde, coniferaldehyde, and sinapaldehyde into *p*- coumaryl-, coniferyl-, and sinapyl-alcohol, respectively, in the lignin biosynthesis pathway (Sibout et al., 2005; Li et al., 2012; Jun et al., 2017). To test whether GhCAD35, GhCAD45, and GhCAD43 have CAD catalytic activities, we generated recombinant GhCAD35, GhCAD45, and GhCAD43 fusion proteins with a GST tag (Figure 4D), incubated individual recombinant GhCAD35, GhCAD45, or GhCAD43 protein with each of the three substrates, *p*-coumaraldehyde, coniferaldehyde, and sinapaldehyde *in vitro*, and analyzed the final reaction products using HPLC. As shown in Figures 4A–C, GhCAD35, GhCAD45, and GhCAD43 all clearly showed CAD catalytic activities to efficiently catalyze *p*-coumaraldehyde, coniferaldehyde, and sinapaldehyde into their corresponding alcohol products, *p*- coumaryl-, coniferyl-, and sinapyl-alcohol, respectively.

To further address the question of the substrate preference and catalytic efficiency, we determined the K_*m*_ and V_*max*_ values of GhCAD35, GhCAD45, and GhCAD43 for the three substrates *p*-coumaraldehyde, coniferaldehyde, and sinapaldehyde, respectively. The three enzymes displayed slight differences in their kinetic parameters (Figure 4E). GhCAD45 generally showed a less affinity toward all the three substrates compared with GhCAD35 and GhCAD43. GhCAD35 showed a little higher affinity toward *p*-coumaraldehyde and sinapaldehyde than coniferaldehyde. GhCAD43 showed a little higher affinity toward coniferaldehyde and sinapaldehyde than p-coumaraldehyde; while GhCAD45 showed no clear affinity preference toward the three substrates (Figure 4E). The three enzymes showed no significant differences in their V_*max*_ values, although GhCAD45 generally showed higher catalytic rates toward all the three substrates compared with GhCAD35 and GhCAD43 (Figure 4E).

Together, these results demonstrate that GhCAD35, GhCAD45, and GhCAD43 have significant CAD catalytic activities to generate monolignol products.

### Silencing *GhCAD35*, *GhCAD45*, or *GhCAD43* Enhances Susceptibility to *V. dahliae*

To investigate whether *GhCAD35*, *GhCAD45*, and *GhCAD43* are involved in *Verticillium* wilt resistance, we used the tobacco rattle virus (TRV)-based VIGS to knock down the expression of individual gene in the *Verticillium* wilt resistant *G. hirsutum* cultivar ZZM 2. The *TRV:CLA* construct was used as a positive control to validate the gene silencing system. As expected, *TRV:CLA* plants displayed a clear bleaching phenotype from the first pair of true leaves (Supplementary Figure 5A), indicating the VIGS system used here was effective and reliable. Next, we compared the expression level of *GhCAD35*, *GhCAD45*, and *GhCAD43* in their corresponding VIGS plants with those in *TRV:00* plants 2 weeks after *Agrobacterium* infiltration by qRT-PCR. The average expression level of *GhCAD35*, *GhCAD45*, and *GhCAD43* in their corresponding VIGS plants was 21.3, 17.4, and 13.0% relative to that in *TRV:00*, respectively, indicating a successful silencing of the target genes (Supplementary Figures 5B,C). In addition, to check the specificity of the gene silencing in the VIGS plants, we also compared the expression of another three most homologous *GhCAD*s of *GhCAD35*, *GhCAD45*, and *GhCAD43*, respectively, in the corresponding *TRV:CAD35*, *TRV:CAD45* and *TRV:CAD43* VIGS plants relative to those of *TRV:00*. As shown in Supplementary Figure 6, the expression of detected *GhCAD*s were not altered in all the VIGS plants, suggesting *TRV:GhCAD35*, *TRV:GhCAD45*, and *TRV:GhCAD43* plants specifically silenced the expression of the target gene, respectively.

**FIGURE 5:**
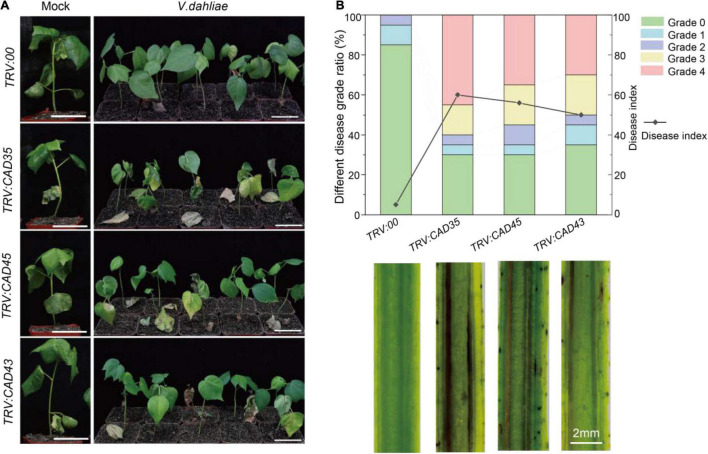
Silencing of *GhCAD35*, *GhCAD45*, and *GhCAD43* in Zhongzhimian 2 (Resistant to *V. dahliae*) resulted in susceptible to *V. dahliae*. **(A)** Phenotypes of the *TRV:00*, *TRV:CAD35*, *TRV:CAD45*, and *TRV:CAD43* VIGS plants at 18 days post *V. dahliae* infection (dpi). **(B)** Ratio of different diseased grades and disease index for *TRV:00*, *TRV:CAD35*, *TRV:CAD45*, and *TRV:CAD43* VIGS plants at 18 dpi (upper panel); the images showing the longitudinal stem sections of *TRV:00*, *TRV:CAD35*, *TRV:CAD45*, and *TRV:CAD43* VIGS plants 18 dpi (lower panel). Two-weeks-old VIGS-plants were inoculated with *V. dahliae* at an inoculum of 1 × 10^7^ spores/mL using the root-dipping method. *n* = 20.

To investigate the responses of gene silencing to *V. dahliae* infection, *V. dahliae* was inoculated in the *TRV:00*, *TRV:GhCAD35*, *TRV:GhCAD45*, and *TRV:GhCAD43* plants 2 weeks after *Agrobacterium* infiltration. The disease symptoms were examined at 18 dpi. As shown in Figure 5A, very weak disease symptoms were observed in *TRV:00* plants; while obvious and typical disease symptoms of *Verticillium* wilt, including leaf chlorosis, wilting, and defoliation were observed in *TRV:GhCAD35*, *TRV:GhCAD45*, and *TRV:GhCAD43* plants. We further quantified the ratio of different disease-grades and disease index (DI) to evaluate the degree of *Verticillium* wilt. Half or more of the *TRV:GhCAD35* (60%), *TRV:GhCAD45* (55%), and *TRV:GhCAD43* (50%) plants displayed the severe grades 3 and 4, while no *TRV:00* plant of these grades were observed and only 15% of *TRV:00* plants displayed the weak grades 1 and 2 (Figure 5B). In addition, *TRV:GhCAD35* plants (DI: 60.0) displayed slightly more severe disease symptoms compared with *TRV:GhCAD45* (DI: 56.3) and *TRV:GhCAD43* (DI: 50.0) plants (Figure 5B). Furthermore, we compared vascular browning of *TRV:00*, *TRV:CAD35*, *TRV:CAD45*, and *TRV:CAD43* plants using longitudinal sections of stems. The vascular tissues of *TRV:GhCAD45* and *TRV:GhCAD43* plants, especially *TRV:GhCAD35*, displayed obviously dark-brown, typical symptom of *V. dahliae* infection, while no such symptom was observed in *TRV:00* plants (Figure 5B). These results indicate that silencing *GhCAD35*, *GhCAD45*, or *GhCAD43* compromised the resistance to *V. dahliae* infection in ZZM 2.

In addition, we chose *GhCAD8* and *GhCAD1*, which were repressed by *V. dahliae* infection in most cultivars 18 dpi (Supplementary Figure 4), and analyzed their roles in resistance to *V. dahliae* infection by examining their VIGS plants. Compared to *TRV:00* plants, *TRV:GhCAD8* plants displayed slight susceptibility to *V. dahliae* infection, and *TRV:GhCAD1* plants showed no *Verticillium* wilt disease symptoms as indicated by plant growth, quantification of disease grades and DI, and observation of vascular tissues 18 dpi (Supplementary Figure 7). Together, these results suggest that *GhCAD35*, *GhCAD45*, and *GhCAD43* are positively involved in *Verticillium* wilt resistance in *G. hirsutum*.

### Silencing *GhCAD35*, *GhCAD45*, or *GhCAD43* Impairs Defense-Induced Lignification in Stems

**FIGURE 6:**
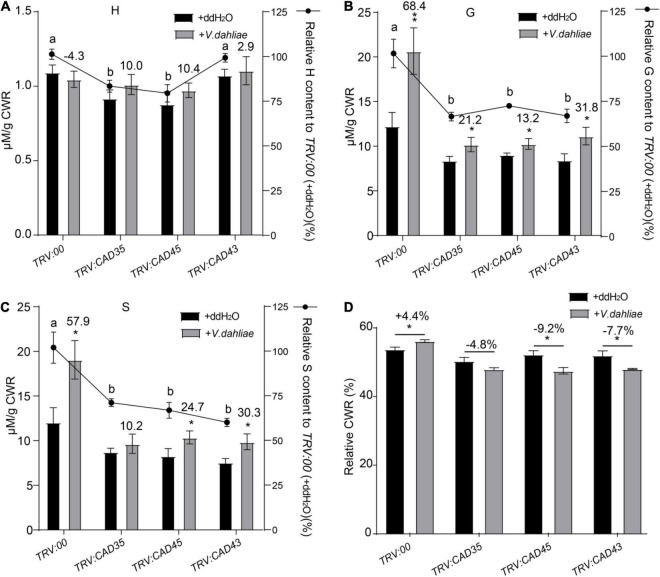
Quantitative analyses of H-, G-, and S-units of lignin in the stem tissues from *TRV:00*, *TRV:CAD35*, *TRV:CAD45*, and *TRV:CAD43* VIGS plants under the control (ddH_2_O) and *V. dahliae* treatment conditions. **(A–C)** Quantitative analyses of H-, G-, and S-units of lignin in *TRV:00*, *TRV:CAD35*, *TRV:CAD45*, and *TRV:CAD43* VIGS plants, respectively. The y axel on the left indicates the contents of individual unit; the y axel on the right indicates the relative content of individual unit, *TRV:CAD35*, *TRV:CAD45*, and *TRV:CAD43* to that of *TRV:00.* The number on top of the gray bars indicates the increase percentage of each unit under *V. dahliae* infection relative to the control (ddH_2_O); *P < 0.05, **P < 0.01 (Student’s *t*-test). The different letters above the black bars indicate significant differences; *P* < 0.05 (Student’s *t*-test). **(D)** The relative cell wall residue (CWR) in *TRV:00*, *TRV:CAD35*, *TRV:CAD45*, and *TRV:CAD43* VIGS plants under the control (ddH_2_O) and *V. dahliae* treatment conditions. The number on top of the Ebars indicates the increase percentage of each unit under *V. dahliae* infection relative to the control (ddH_2_O); *P < 0.05 (Student’s *t*-test); the data represent the mean ± SD (*n* = 3 biological replicates). Two-weeks-old VIGS-plants were inoculated with *V. dahliae* at an inoculum of 1 × 10^7^ spores/mL using the root-dipping method. The samples were collected 48 h after the inoculation.

*Verticillium dahliae* induced lignification has been reported to confer resistance to *Verticillium* wilt in plants (Smit and Dubery, 1997; Gayoso et al., 2010). To evaluate the involvement of *GhCAD35*, *GhCAD45*, and *GhCAD43* in lignin biosynthesis, we quantitatively measured the composition of the three lignin monomers, i.e., H-, G-, and S-unit, assayed by thioacidolysis and analyzed by gas chromatography mass spectrometry (GC-MS) using stems of *TRV:00*, *TRV:GhCAD35*, *TRV:GhCAD45*, and *TRV:GhCAD43* plants with or without infection by *V. dahliae* (48 h after inoculation). In *TRV:00* plants, *V. dahliae* infection significantly induced lignin biosynthesis and deposition (+60.0%), mainly the G- and S-unit lignin (+68.4% and +57.9%, respectively) (Figures 6B,C). Compared to *TRV:00* plants, *TRV:GhCAD35*, *TRV:GhCAD45*, and *TRV:GhCAD43* plants showed a decrease of the total lignin content, mainly due to decreased G- and S-unit, especially under *V. dahliae* infected condition (Supplementary Table 1). In addition, the levels of the G-unit and S-unit were increased in *V. dahliae* infected *TRV:GhCAD35*, *TRV:GhCAD45*, and *TRV:GhCAD43* plants compared to their corresponding control plants, despite not as significant as that in *TRV:00* plants (Figures 6B,C). No significant difference was observed for the effect of *V. dahliae* infection on the H-unit lignin between VIGS plants and controls (Figure 6A). Cell wall residue (CWR) mainly indicates the ratio of lignin in the whole tissue. Notably, CWR showed a slight but significant increase in *TRV:00* plants after *V. dahlia* inoculation; in contrast, a decrease trend of CWR was observed in the *V. dahliae* infected *TRV:GhCAD35*, *TRV:GhCAD45*, and *TRV:GhCAD43* plants compared to their corresponding controls (Figure 6D).

**FIGURE 7:**
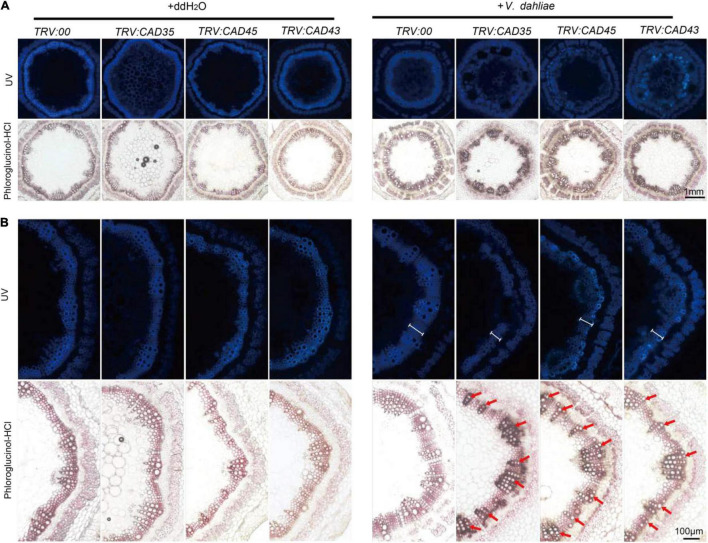
Histochemical analyses of lignin in the stem cross-sections from *TRV:00*, *TRV:CAD35*, *TRV:CAD45*, and *TRV:CAD43* VIGS plants at 18 dpi. **(A)** The handcut stems were illuminated by UV or stained with phloroglucinol-HCl to detect lignin. **(B)** Close-up of UV autofluorescence and phloroglucinol-HCl stain of stems corresponding to images in A. Two-weeks-old VIGS plants were inoculated with *V. dahliae* at an inoculum of 1 × 10^7^ spores/mL using the root-dipping method. Scale bars in A and B represent 1 mm and 100 μM, respectively. The white scales in *V. dahliae* inoculated stems under UV indicate the width of xylem; the red arrows indicate the vessels.

To further examine the effects of *GhCAD35*, *GhCAD45*, and *GhCAD43* on lignin deposition upon *V. dahliae* infection, we sectioned the stems of the *TRV:00*, *TRV:GhCAD35*, *TRV:GhCAD45*, and *TRV:GhCAD43* plants at 18 dpi, and checked deposition of lignin in xylem based on the autofluoresence under UV light and by phloroglucinol-HCl staining. Under the control (ddH_2_O) conditions, no difference in lignin deposition was observed in stems among *TRV:00*, *TRV:GhCAD35*, *TRV:GhCAD45*, and *TRV:GhCAD43* plants (Figure 7); however, under the *V. dahliae* infection conditions, the xylem width of the stems from the *TRV:GhCAD35*, *TRV:GhCAD45* and *TRV:GhCAD43* plants was much thinner compared to that of *TRV:00* indicated by autofluorescence under UV illumination and the color stained by phloroglucinol-HCl (Figure 7). Moreover, the *V. dahlia* infection could be observed surrounding the vessels in the *V. dahlia* infected VIGS plants, indicating spreading of the *V. dahlia* infection from the root to the shoot through vessels (Figure 7). These results were further confirmed by the quantification of the lignin composition of H-, G-, and S-unit. As shown in Table 1, under control (ddH2O) conditions, the contents of the three units in *TRV:GhCAD35*, *TRV:GhCAD45*, and *TRV:GhCAD43* plants were slightly lower than that of *TRV:00*; however, upon *V. dahliae* infection, all the three units significantly reduced at 18 dpi in *TRV:GhCAD35*, *TRV:GhCAD45*, and *TRV:GhCAD43* compared with *TRV:00* plants. Moreover, the reduction of the S unit was more severe than the H- and G-units, leading to a significantly reduced S/G ratio in *TRV:GhCAD35*, *TRV:GhCAD45*, and *TRV:GhCAD43* plants but not in *TRV:00* plants (Table 1). In addition, to check whether the silencing of *GhCAD* genes disrupts the expression of phenylpropanoid metabolic and lignin biosynthesis genes, we checked the expression of several genes in phenylpropanoid metabolic pathway, such as *GhPAL1, Gh4CL2, GhHCT, GhCOMT1, GhCCR1*, etc. However, no clear induction or repression was observed for the investigated genes (Supplementary Figure 8).

**TABLE 1:** Quantification of H-, G-, and S-unit of lignin deposition in stems of *TRV:00*, *TRV:CAD35*, *TRV:CAD45*, and *TRV:CAD43* VIGS plants at 18 dpi by *V. dahliae*.

	Sample name	Monomer yields			Total yields	Molar ratios
		H	G	S		S/G
+ddH_2_O	*TRV*:*00*	5.5 ± 0.9	67.8 ± 9.5	66.7 ± 8.3	140	0.98
	*TRV*:*CAD35*	4.6 ± 0.5	59.5 ± 7.2	61.5 ± 8.8	125.6	1.03
	*TRV*:*CAD45*	6.1 ± 0.3	55.6 ± 5.0	57.3 ± 5.2	119	1.03
	*TRV*:*CAD43*	4.7 ± 0.2	61.8 ± 6.0	58.5 ± 9.7	125	0.95
+*V. dahliae*	*TRV*:*00*	5.1 ± 0.2	62.1 ± 4.9	46.9 ± 5.2	114.1	0.76
	*TRV*:*CAD35*	5.0 ± 0.2	52.0 ± 8.8	25.0 ± 4.5	75	0.48
	*TRV*:*CAD45*	5.5 ± 0.7	51.9 ± 4.7	31.2 ± 3.6	88.6	0.6
	*TRV*:*CAD43*	5.9 ± 0.6	55.5 ± 7.0	39.2 ± 5.3	100.6	0.71

*Yields (μmol/g of Klason Lignin) of Lignin-Derived Thioacidolysis Monomers from Various Lignocellulosic Materials, Measured by GC–MS.*

*H, p-hydroxyphenyl monomer; G, guaiacyl monomer; S, syringyl monomer.*

Taken together, these results demonstrate *GhCAD35*, *GhCAD45*, and *GhCAD43* positively regulate *V. dahliae* induced lignification in the stems of Upland cotton.

### Silencing *GhCAD35*, *GhCAD45*, or *GhCAD43* Alters *V. dahliae* Induced Salicylic Acid Responses

**FIGURE 8:**
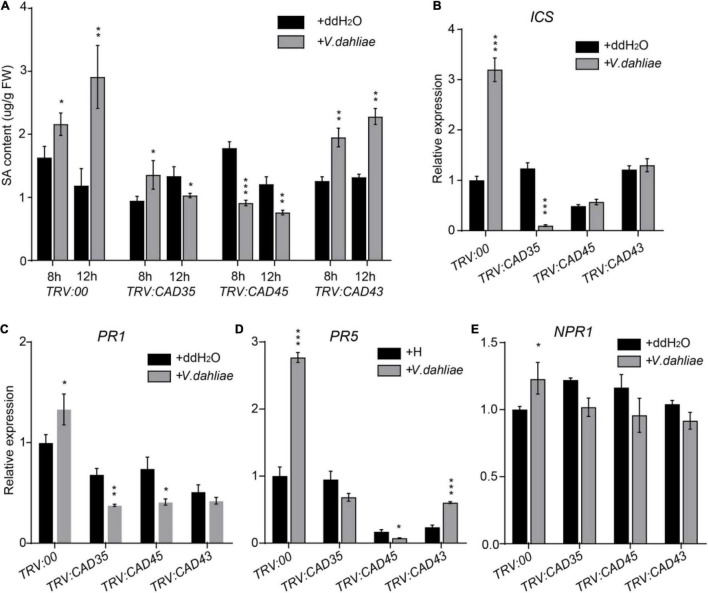
Silencing of *GhCAD35*, *GhCAD45*, and *GhCAD43* impaired *V. dahliae* induced SA responses. **(A)** Quantification of SA contents in *TRV:00*, *TRV:CAD35*, *TRV:CAD45*, and *TRV:CAD43* VIGS plants 8- and 12-h post-infection by *V. dahliae*. **(B–E)** The expression level of SA biosynthesis gene *ICS* and SA responsive genes *PR1*, *PR5*, and *NPR1* in *TRV:00*, *TRV:CAD35*, *TRV:CAD45*, and *TRV:CAD43* VIGS plants 12-h post-infection by *V. dahliae*. The data represent the mean ± SD (*n* = 3 biological replicates), *P < 0.05, **P < 0.01, ***P < 0.001 (Student’s *t*-test).

Salicylic acid and JA play key roles in plant defense against pathogen infection (Shaban et al., 2018; Song et al., 2020). Previous studies have shown that disturb of lignin metabolism in the cell wall of root and stem tissues in *G. hirsutum* results in the change of SA synthesis and signaling upon *V. dahliae* infection. Therefore, we investigated the effects of silencing *GhCAD35*, *GhCAD45*, or *GhCAD43* on the SA biosynthesis and signaling. As shown in Figure 8A, SA content was significantly increased in *TRV:00* plants 8-h- and 12-h-post inoculation of *V. dahliae* (Figure 8A); however, SA contents were lower or less induced in the *TRV:GhCAD35*, *TRV:GhCAD45*, and *TRV:GhCAD43* plants compared to that of *TRV:00* plants after *V. dahliae* infection. We further investigated the expression of SA biosynthesis gene *ICS1*, and SA signaling genes *PR1*, *PR5*, and *NPR1* 12-h-post *V. dahliae* infection by qRT-PCR. Consistently, the expression levels of *ICS1*, *PR1*, *PR5*, and *NPR1* were all induced by *V. dahliae* infection in *TRV:00* plants; while in *TRV:GhCAD35* and *TRV:GhCAD45*, the expression levels of *ICS1*, *PR1*, and *PR5* were inhibited or no significant change after *V. dahliae* infection. In *TRV:GhCAD43*, a less induction or no significant change were observed for the expression of *ICS*, *PR1*, and *PR5* after *V. dahliae* infection compared to those of *TRV:00* plants (Figures 8B–D). We also investigated the effects of silencing *GhCAD35*, *GhCAD45*, or *GhCAD43* on the gene expression of JA signaling components, such as *GhJAZ3, GhJAZ10, GhMYB2-2*, and *GhMYB2-3*. As shown in Supplementary Figure 9, *GhJAZ10* was slightly induced after *V. dahliae* infection in *TRV:GhCAD35* and *TRV:GhCAD45*, but the other genes did not show consistent changes in the *GhCAD* silencing plants after *V. dahliae* infection compared to that of control. These results indicate that silencing *GhCAD35*, *GhCAD45*, or *GhCAD43* might affect the defense induced SA responses at the early infection stage.

### Heterogeneous Overexpression of *GhCAD35*, *GhCAD45*, or *GhCAD43* in *Arabidopsis* Enhances Resistance to *V. dahliae*

**FIGURE 9:**
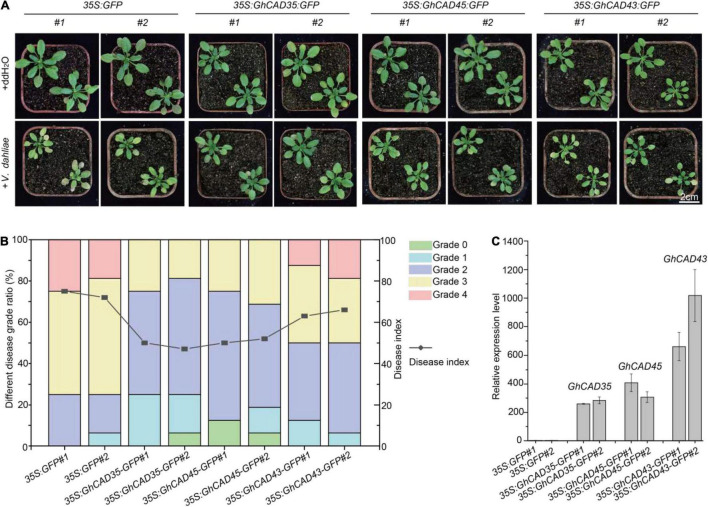
Heterogeneously expressing of *GhCAD35, GhCAD45*, and *GhCAD43* in *Arabidopsis* enhanced resistance to *V*. *dahliae* infection. **(A)** Phenotypes of the transgenic *Arabidopsis* plants overexpressing *GhCAD35*, *GhCAD45*, or *GhCAD43* at 14 days post-infection (dpi) by *V. dahliae*. **(B)** Ratio of different diseased grades and disease index for *GhCAD35*, *GhCAD45*, and *GhCAD43* overexpressing transgenic *Arabidopsis* plants at 14 dpi by *V. dahliae.* Two independent Arabidopsis transgenic lines of *35S:GhCAD35-GFP*, *35S:GhCAD45-GFP*, and *35S:GhCAD43-GFP* were used for *V. dahliae* infection. *35S:GFP Arabidopsis* transgenic plants were used as control. Two-weeks-old *Arabidopsis* transgenic plants were inoculated with *V. dahliae via* root-dipping method; 16 plants for each line were used. **(C)** The expression level of *GhCAD35, GhCAD45*, and *GhCAD43* in the corresponding Arabidopsis transgenic plants determined by qRT-PCR.

The above results indicate that *GhCAD35*, *GhCAD45*, and *GhCAD43* are positive regulators of resistance to *V. dahliae* by affecting lignin biosynthesis and deposition. To further confirm this conclusion, we heterogeneously overexpressed *GhCAD35*, *GhCAD45*, or *GhCAD43* in *Arabidopsis* to examine their effects on *V. dahliae* infection. We analyzed two independent *Arabidopsis* transgenic lines overexpressing *GhCAD35*, *GhCAD45*, or *GhCAD43*, and challenged the transgenics with *V. dahliae*. As shown in Figures 9A–C, compared to the control *35S:GFP* plants, all *Arabidopsis* transgenic lines displayed overexpressed expression of corresponding *GhCAD*s and enhanced resistance to *V. dahliae* infection, as indicated by plant growth, disease infection grades, and disease index statistics, although the resistance of the transgenics overexpressing *GhCAD43* was not as significant as those of the transgenes overexpressing *GhCAD35* or *GhCAD45*. Furthermore, we chose one line of each *GhCAD*s overexpressing transgenic *Arabidopsis* plants and measured the H-, G-, and S-units of lignin contents in their stems. As shown in Supplementary Figure 10, S-unit of lignin was significantly elevated in the *GhCAD*s overexpressing transgenic *Arabidopsis* plants compared with that of control, suggesting overexpression of *GhCAD35*, *GhCAD45* or *GhCAD43* enhanced the lignin biosynthesis in Arabidopsis.

## Discussion

As one key component of plant cell wall, lignin is important for plant immunity and defense responses (Xie et al., 2018; Cesarino, 2019; Dong and Lin, 2021). The lignin deposition in the cell wall provides the first barrier for pathogen invasion (Dong and Lin, 2021). In addition, the induced lignification by pathogen invasion further inhibits the growth, movement and infection ability of the pathogens (Xie et al., 2018; Li et al., 2020). In cotton, lignin biosynthesis is significantly induced upon *V. dahliae* infection and has a significant role in resistance to *V. dahliae* (Zhu et al., 2018; Li et al., 2020; Xiao et al., 2021; Xiong et al., 2021). Therefore, it is important to identify the genes responsible for the *V. dahliae* induced lignification. The *CAD* gene family encode a class of NADPH-dependent enzymes involved in the last step of monolignol biosynthesis, therefore important for lignin deposition (Kim et al., 2007; Dong and Lin, 2021). Previously study has identified 20 *GhCAD*s in *G. hirsutum*, which are divided into three groups based on homology analysis with the nine *bona fide Arabidopsis CADs* (Zhang S. et al., 2020). In the present study, we reexamined the *CAD* gene family in *G. hirsutum* and identified 46 *GhCAD* or *GhCAD-like* genes that were classified into five groups (Figure 1A). By comparing the response of *GhCADs* to *V. dahliae* infection in cultivars resistant or susceptible to *V. dahliae*, *GhCAD35*, *GhCAD45*, and *GhCAD43* were selected for further functional characterization.

The *in vitro* enzymatic assay showed that GhCAD35, GhCAD45, and GhCAD43 are active enzymes for catalyzing *p*-coumaraldehyde, coniferaldehyde, and sinapaldehyde into *p*- coumaryl-, coniferyl-, and sinapyl-alcohol, respectively (Figure 4), with no obvious enzyme kinetic difference. Notably, GhCAD43 is a member of the group V CAD-like protein (Figure 1A), suggesting a role of *CAD-like* genes in biosynthesis of lignin *in planta*.

*Verticillium dahliae* induced lignin biosynthesis has been shown in various plant species, such as pepper, tomato, and cotton (Pomar et al., 2004; Gayoso et al., 2010; Xiong et al., 2021). Consistently, we observed significantly elevated G-unit and S-unit in *V. dahliae* infected Upland cotton cultivar (ZZM 2) resistant to *V. dahliae*, but the *V. dahliae* induced lignin deposition was compromised in VIGS plants of *GhCAD35*, *GhCAD45*, or *GhCAD43* (Figure 6 and Supplementary Table 1). Consequently, the *TRV:GhCAD35*, *TRV:GhCAD45*, and *TRV:GhCAD43* plants were more susceptible to *V. dahliae* compared to *TRV:00* (Figure 5). At the latter infection stage (18 dpi), lignin deposition was decreased in all *V. dahliae* infected plants compared to those of uninfected, which could attribute to the breakdown of lignin upon *Verticillium* wilt disease spreading (Figure 7). These results suggest *GhCAD35*, *GhCAD45*, and *GhCAD43* play a pivotal role in *V. dahliae* induced lignification and resistance to the pathogen.

The biosynthesis of lignin mainly results from the oxidative coupling of three monolignols, giving rise to G-, H-, and S-unit, respectively. The proportions of these three units in the cell wall are significantly diverse depending on plant species, developmental stages, and tissue types (Bhuiyan et al., 2009). Plants have adopted an effective mechanism to restrict the spread of pathogen, by restructuring the monomer composition of lignin in the vascular tissues (Pomar et al., 2004; Gayoso et al., 2010). Generally, the lignin in cotton stems contains much higher proportions of G- and S-unit than H-unit (Xiong et al., 2021). Consistently, our study showed that G- and S-unit were predominant compared to H-unit in cotton stems (Table 1 and Supplementary Table 1). In addition, it was reported that G-rich lignin has a more pronounced impact on cotton defense responses (Xu et al., 2011; Zhang et al., 2019). However, several studies also indicate a similar role of S-rich lignin in plant immunity (Wang et al., 2018). Our study showed that the contents of both G- and S-unit were significantly induced 48 h after *V. dahliae* infection, with a slight higher induction of G- than S-unit (+68.4% vs. +57.9%), indicating both G- and S-unit play pivotal roles in the early defense responses (Figure 6 and Supplementary Table 1). However, at the latter infection stage, the S/G ratio greatly decreased in the *V. dahliae* infected plants compared to that of control (Table 1), suggesting that G-rich lignin might play a more pronounced role for resistance at the latter infection stage.

The Upland cotton genome contains 46 *GhCAD* genes, how do they coordinate with each other to regulate lignin biosynthesis is an interesting question. Here, we showed that silencing a single *GhCAD* (*GhCAD35*, *GhCAD45*, or *GhCAD43*) seems to be sufficient to repress *V. dahliae* induced lignin biosynthesis (Figure 5), suggesting they likely act synergistically rather than redundantly in lignin biosynthesis. The tempo-spatial expression of genes provides key and important insights for their biological functions. Most *GhCADs* are expressed in the root, leaf, and stem tissues at different developmental stages (Figure 1B). Interestingly, *GhCAD35* and *GhCAD45* belong to the mediate- and low-expression subgroup, respectively; while *GhCAD43* displayed an obvious developmental stage dependent manner with low expression at seedling stage but high expression at the latter developmental stage (Figure 1B). Therefore, the induction of their expression by *V. dahliae* infection indicates *GhCAD35*, *GhCAD45*, and *GhCAD43* are tightly regulated by internal and external factors. Transcriptional factors (TFs) usually act as master switches by controlling gene expression under different developmental or environmental conditions. Numerous TFs have been illustrated to be involved in plant lignin biosynthesis, including many members of the *MYB* family (Zhong et al., 2008; Zhou et al., 2009; Dong and Lin, 2021). TFs from the *WRKY*- and *NAC*-family are also involved in the regulation of lignin biosynthesis (Wang et al., 2020; Dong and Lin, 2021; Hu et al., 2021). Therefore, it is important to study how the expression of *GhCADs* is regulated by these TFs in the future.

Phytohormones such as abscisic acid, jasmonate acid, ethylene, and SA, play critical roles in the complex defense responses (Bari and Jones, 2009). Previous studies have shown that the alteration of lignin metabolism in the cell wall of root and stem tissues in *G. hirsutum* results in the change of SA synthesis and signaling upon *V. dahliae* infection (Li et al., 2019; Tang et al., 2019). Here we showed that the expression level of the SA biosynthesis gene *ICS1*, and the SA signaling genes *PR1 PR5*, and *NPR1* were altered in *TRV:GhCAD35*, *TRV:GhCAD45*, and *TRV:GhCAD43* plants compared to *TRV:00* (Figure 8). How does silencing *GhCAD*s affect SA content upon *V. dahliae* infection is still an open question. The cross-talk between SA and lignin metabolism on fine-tuning the defense response of cotton toward *V. dahliae* infection remains to be determined. We speculate that silencing *CAD*s results in impaired defense responses, which might indirectly affected the SA levels upon *V. dahliae* infection. On the other hand, *GhCAD35*, *GhCAD45*, and *GhCAD43* catalyze the generation of various monolignols, which likely affects the turnover of intermediates in lignin biosynthesis pathway that might act as phytoalexins or signal molecules in the plant defense responses (Dixon et al., 2002). One of the main goals of studying defense-induced lignification is to utilize the generated knowledge to develop crops with improved tolerance to *V. dahliae*. Future work by modulating the expression of *GhCADs* involved in lignin biosynthesis through bioengineering or molecular breeding strategies might be able to develop cotton cultivars resistant to *Verticillium* wilt.

## Experimental Procedures

### *Gossypium hirsutum* and *Arabidopsis* Growth Conditions

Nine Upland cotton (*G. hirsutum* L.) accessions, including four (Zhongzhimian 2, Han 333, GK 44, and GK 164) resistant to *V. dahliae* and five (Jimian 11, DH 966, Foster 6, 8891, and TM-1) susceptible to *V. dahliae*, were used in disease assay. Plants were grown at 25°C in a growth room with 80–100 μmol⋅m^–2^⋅s^–1^ light and a 16-hr light/8-hr dark photoperiod. Two-weeks-old cotton plants were used in *V. dahliae* infection with the strain of V991.

The *V. dahliae* resistant cultivar Zhongzhimian 2 (ZZM 2) was used in the VIGS experiment and disease assay. The experiment was performed in a growth room with a temperature of 23–24°C, light intensity of 100 μmol⋅m^–2^⋅s^–1^, and a photoperiod of 16-hr light/8-hr dark.

The *Arabidopsis* (Col-0 background) plants were grown at 22°C in a growth chamber under 100 μmol⋅m^–2^⋅s^–1^ light and a 16-hr light/8-hr dark photoperiod. *Arabidopsis* transformation was performed using the flower dipping method as described previously (Clough and Bent, 1998).

### Identification of the *CAD* Genes in *G. hirsutum*

Two approaches were adopted to identify cotton *CAD* genes in *G. hirsutum*. First, nine bona fide *Arabidopsis* CAD proteins were used to search Arabidopsis TAIR database (see text footnote 1) and identified another nine CAD-like proteins in *Arabidopsis* based on domain analysis. *Arabidopsis* CAD and CAD-like protein sequences were used as queries to search against the annotated proteins of *G. hirsutum* (*Gossypium hirsutum* (AD1) ‘TM-1’ genome NAU-NBI_v1.1_a1.1) (Zhang et al., 2015) downloaded from the CottonGen database^2^ by local blast tool with the default settings (E-value < 1*^e–10^*). All hits were considered as potential candidate *CAD* genes. Second, the hidden Markov model (HMM) profile of the two conserved domains (PF08240, alcohol dehydrogenase GroES-like domain; PF00107, zinc-binding dehydrogenase)^3,4^ (Potter et al., 2018) was used to search the *G. hirsutum* (*Gossypium hirsutum* (AD1) ‘TM-1’ genome NAU-NBI_v1.1_a1.1) (Zhang et al., 2015) using the HMMER 3.0 software. All candidate sequences derived from the two searches were subjected to domain analysis using the InterProScan^5^ and SMART^6^ tools with the default parameters. Only protein sequences with both domains were considered to be CADs. The gene ID and corresponding gene name for all *GhCAD*s are listed in Supplementary Table 2.

### *Verticillium dahliae* Inoculation

The *V. dahliae* strain V991 was cultivated on potato dextrose agar (PDA) plate at 25°C for 4–5 days, and was then transferred to potato dextrose broth (PDB) liquid media and incubated on a shaker (120 rpm, 25°C) for 3 days. The conidia of *V. dahliae* were collected and adjusted to a concentration of ∼1 × 10^7^ spores/mL. Cotton seedlings were inoculated by root dipping with the inoculum. Three replicates were set up for each genotype with at least 20 plants in each replicate.

### Disease Index Calculation

Disease index was calculated according to the method descripted previously (Miao et al., 2019). Disease symptom severity was scored as level 0 to level 4 at 18 dpi according to the percentage of wilted true leaves and leaf chlorosis or defoliation. A score of zero means no visible wilting or chlorosis symptoms; a score of 1–4 indicates 0–25% (inclusive), 25–50% (inclusive), 50–75% (inclusive), and 75–100% of wilted true leaves and leaf chlorosis or defoliation, respectively. DI was calculated according to the following formula:


$$\text{DI}=\frac{\text{Sum}\,\left(n\,\mathrm{number}\,\mathrm{of}\,\mathrm{plants}\,\mathrm{at}\,\mathrm{level}\,n\right)}{4\times \mathrm{the}\,\mathrm{total}\,\mathrm{number}\,\mathrm{of}\,\mathrm{plants}}\times 100$$

*n* represents disease levels ranging from 0 to 4.

### RNA Isolation and Quantitative Real-Time PCR Analysis

Total RNA was extracted from cotton stems using a RNAprep Pure Plant Plus Kit (Polysaccharides and Polyphenolics-rich) (Tiangen, Beijing, China). Then 1 μg total RNA was taken for reverse transcription to synthesize cDNA using HiScript II Q Select RT SuperMix (Vazyme, Nanjing, China). The quantitative real-time PCR (qRT-PCR) experiment was conducted using ChamQ SYBR Color qPCR Master Mix (Vazyme, Nanjing, China) and analyzed using the Roche LightCycler 480 (Beijing, China). The thermal profile for qRT-PCR was 95°C for 2 min, followed by 40 cycles of 95°C for 15 s and 60°C for 30 s. Primers used for qRT-PCR are listed in Supplementary Table 3.

### Gene Cloning, Protein Expression and Purification

Full-length *GhCADs* were amplified with a KOD-Plus kit (Toyobo) using the cDNA generated from TM-1. The gene specific primers were designed according to the TM-1 genome sequence of NAU-NBI_v1.1. GhCADs were expressed in *E. coli* (BL21) by cloning individual *GhCAD* into a glutathione (GST)-tagged expression construct *pGEX4T-1*. The recombinant GST-GhCADs were purified using a column containing GST coupled with agarose medium. Western blot was used to detect the recombinant GST-GhCADs using anti-GST antibody on an SDS-PAGE Gel.

### Enzyme Assay and Kinetics

The reaction (200 μL) used in assay of GhCAD activity contains: 50 mM Tris HCl (pH = 7.5), 0.5 mM NADPH, 0.1 mM substrate (coniferyl aldehyde, p-coumaryl aldehyde, or sinapyl aldehyde), 2.0 mM dithiotreitol (DTT), and 1.3 μg recombinant GST-GhCAD protein. The reaction was incubated at 30°C for 30 min and terminated by adding 200 μL methanol. The reaction mixture was filtrated through a 0.22 μm organic diameter filter. A volume of 100 μL filtrated reaction mixture was then subjected to high-performance liquid chromatograph (HPLC) analysis with an XBridge BEH C18 column (2.5 μm, 3.0 × 150 mm XP). The experimental procedure established to separate the products (three alcohols) from the substrates (three aldehydes) was as follows: two chromatographic grade solvents, water (containing 1% formic acid) and acetonitrile, were used as the mobile phase A and B, respectively; the proportion of A:B was set to change from 95:5 to 5:95 from 0 to 30 min gradually; the flow rate was set to be 0.5 mL/min; the section of full wavelengths was set to be from 210 nm to 400 nm.

In order to analyze the GhCAD catalytic kinetics, nine concentrations (2, 6, 8, 17, 25, 33, 42, 50, and 100 μM) of each substrate were chosen to conduct the catalytic experiment as mentioned above, and the enzyme kinetic curves were fitted using the Origin 2018 software (Pan et al., 2014; Ma et al., 2018).

### Virus-Induced Gene Silencing

A gene-specific fragment encoding 100–200 amino acids was amplified from *GhCAD35, GhCAD43*, or *GhCAD45*, and cloned into the *pTRV2* plasmid between the EcoRI and KpnI restriction sites. Primers used are listed in Supplementary Table 4. After confirmation by sequencing, the recombinant vector was introduced into *Agrobacterium tumefaciens* GV3101 by thermal activation. For VIGS, the *Agrobacterium* culture was resuspended in the VIGS buffer (10 mM MgCl_2_, 10 mM MES and 200 μM acetosyringone) and mixed with *Agrobacterium tumefaciens* transformed with *pTRV1* in equal proportion. After 3 h of incubation at room temperature, various *Agrobacterium* combinations were injected into cotyledons of 1 week old seedlings of ZZM 2. Two weeks later, the treated cotton seedlings were used in confirmation of silencing of the target gene (based on qRT-PCR) and then used in disease assay by inoculation of *V. dahliae* inoculum (as mentioned above).

### Phloroglucinol Staining

Stems collected from the same position of different cotton plants were cross sectioned by hand with a section thickness of 100–120 μM. Sections were immersed in 1% phloroglucinol (0.1 g phloroglucinol in 10 mL 95%EtOH) for 3–5 min; afterward, sections were gently rinsed with water and acidified with 20% HCl solution. Finally, the sections were examined by inverted microscope.

### Cell Wall Preparation

Freeze-dried stem materials were fully ground to fine powder and extracted in 70% EtOH (5 × volume) at 65°C for 3 h. After repeated twice the extraction, the final extraction was carried out with chloroform/methanol (1:1 v/v) to get rid of water and pigments. The products were washed once by acetone (2–3 × volume) and dried for 3 days in an oven in a hood.

### GC-MS Measurement of Lignin G-, S-, and H-Units

Lignin G-, S-, and H-units were depolymerized and separated with a streamlined thioacidolysis, and analyzed by gas chromatography–mass spectrometry (GC–MS) as previously described (Robinson and Mansfield, 2009). For each sample, 10 ± 1 mg of ground, oven-dried wood powder was filled into a 2-mL HPLC glass vial. The vials were blanked with nitrogen gas, added with 1 mL of freshly made reaction buffer [2.5% boron trifluoride etherate and 10% ethanethiol, in recently distilled dioxane (v/v)], and tightly capped. The vials were then placed in a dry heating block (98°C) for 4 h, with periodic manual agitation. The reaction was stopped by placing on ice for 15 min and adjusted to have a pH value between 3 and 4 by adding appropriate volume of 0.4 M sodium bicarbonate (∼0.3 mL, as determined by pH indicator paper). The solution was transferred to a new 10 mL vial. Internal standard (methyl heptadecanoate, 1 mg) dissolved in 1 mL methylene chloride was then added to each vial. To extract the reaction products from the aqueous mixture, the vials were recapped, vortexed and kept still for half an hour, phase-separating the upper (aqueous) and lower (organic, containing lignin breakdown products) phases. An aliquot (1.5 mL) of the organic phase was removed and simultaneously cleared of residual water and filtered by passing through a Pasteur pipette packed with a small tissue-paper plug and an inch (∼50 mg) of granular anhydrous sodium sulfate, and transferred directly into a 2-mL polypropylene microfuge tube. Samples were then collectively evaporated to dryness in a low pressure oven (50°C) overnight and resuspended in 0.5 mL of methylene chloride. A volume of 50 μL samples was transferred to new microcentrifuge tube and dried in the same condition as above; the dried samples were then combined with 50 μL of pyridine and 50 μL of *N*-methyl-*N*-(trimethylsilyl) trifluoroacetamide. After incubation for at least 5 h at room temperature, 1 μL of the reaction product was analyzed by GC-MS.

GC-MS was conducted on gas chromatography tandem mass spectrometry Xevo TQ-GC (Waters, United States). The analyses used were as previously described (Rolando et al., 1992). In brief, the GC-MS was equipped with a DB-5 column (30 m × 0.25 mm × 0.25 μm, Agilent Technologies). The oven initial temperature on the DB-5 column was maintained at 130°C for 3 min and then increased to 250°C at 3°C/min, and finally maintained 250°C for 5 min. The injector temperature was maintained at 250°C with a constant flow rate of 1.0 mL/min of helium. The inlet was operated in pulsed splitless injection mode. The GC–MS electron impact source was operated in scan mode with the MS source temperature at 280°C and MS Quad at 150°C.

### Salicylic Acid Measurement

Endogenous SA was extracted from the stems of cotton plants as previously described (Hu et al., 2018; Miao et al., 2019), and measured by LC-MS. In brief, three replicates, each with 100 mg (fresh weight) of stem samples, were harvested at 8 and 12 h after *V. dahliae* inoculation. The samples were ground to a fine powder in liquid nitrogen, mixed with 750 μL of ice-cold 80% methanol containing 1% acetic acid, and placed on a shaker for 16 h at 4°C in darkness. The samples were then centrifuged at 12,000 rpm (4°C) for 15 min, and the supernatant was collected and transferred into a new tube and was then evaporated to dryness, and the residue was reconstituted in 200 μL of 80% methanol. Finally, the extracts were analyzed using a Q Exactive Plus HPLC-MS system (Thermo, United States).

## Data Availability Statement

The original contributions presented in the study are included in the article/Supplementary Material, further inquiries can be directed to the corresponding author/s.

## Author Contributions

YM, K-PJ, and HL conceived the study. SZ performed bioinformatic analysis. HL, XlZ, WX, YG, and YW performed the other experiments. YM, K-PJ, HL, SZ, and YZ analyzed the data. K-PJ, HL, and SZ wrote the manuscript. YM, YZ, Q-HZ, XeZ, KL, and JG revised the manuscript. All authors contributed to the article and approved the submitted version.

## Conflict of Interest

A patent application about the application of GhCAD35, GhCAD45 and GhCAD43 on Upland cotton breeding has been filed. The authors declare that the research was conducted in the absence of any commercial or financial relationships that could be construed as a potential conflict of interest.

## Publisher’s Note

All claims expressed in this article are solely those of the authors and do not necessarily represent those of their affiliated organizations, or those of the publisher, the editors and the reviewers. Any product that may be evaluated in this article, or claim that may be made by its manufacturer, is not guaranteed or endorsed by the publisher.

## Footnotes

1
www.arabidopsis.org

2
https://www.cottongen.org/home

3
http://Pfam.sanger.ac.uk/

4
http://hmmer.org/

5
http://www.ebi.ac.uk/Tools/pfa/iprscan5/

6
http://smart.embl-heidelberg.de/
