# Supplementary material for: Identification and Characterization of Cinnamyl Alcohol Dehydrogenase Encoding Genes Involved in Lignin Biosynthesis and Resistance to Verticillium dahliae in Upland Cotton (Gossypium hirsutum L.)
Source: Front Plant Sci. 2022 Apr 28;13:840397. doi: 10.3389/fpls.2022.840397 (PMC9096875; doi:10.3389/fpls.2022.840397)
Supplement: Supplementary file 2 [file Data_Sheet_2.docx]

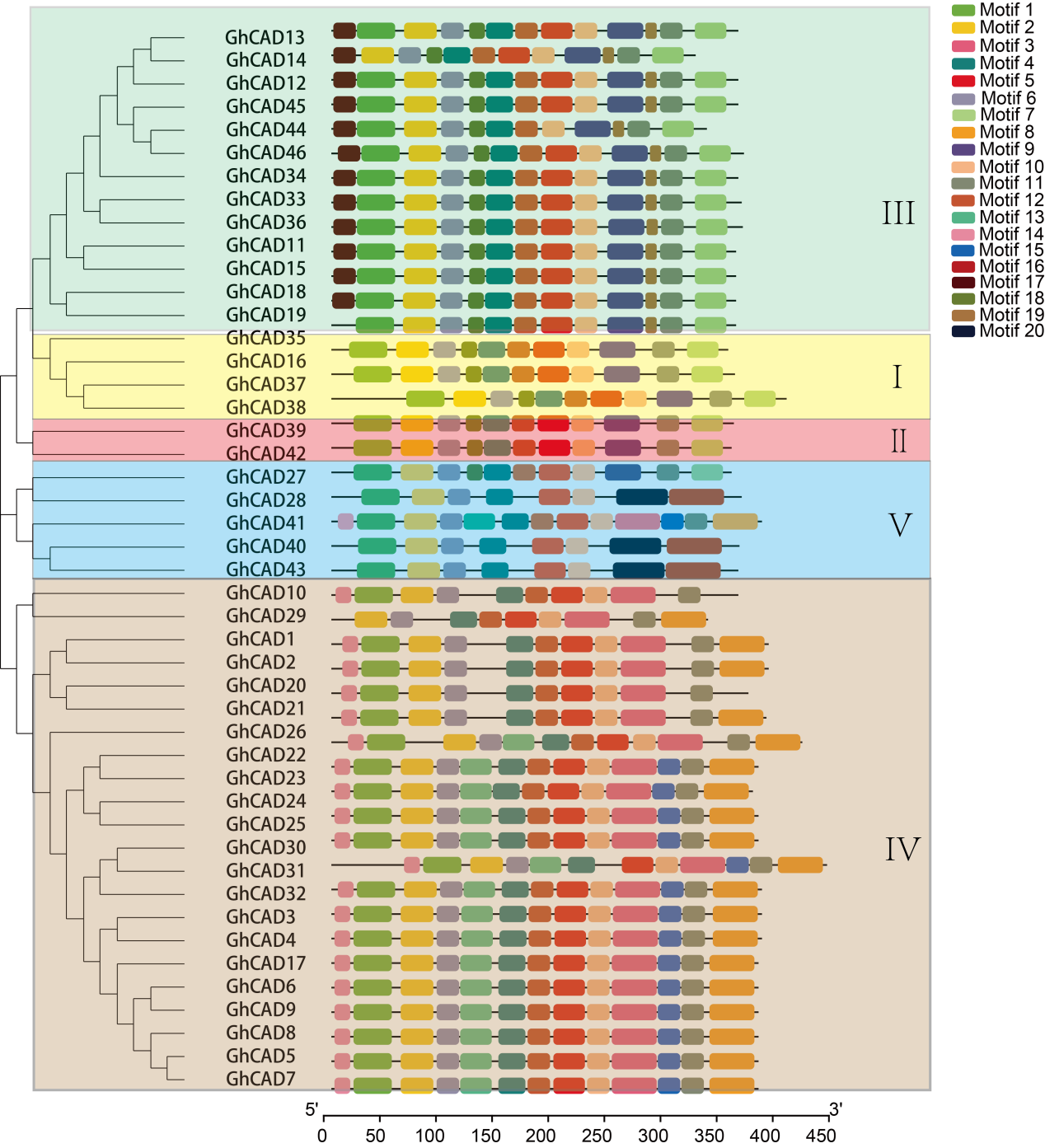


**Figure S1**. Motif structures of the amino acids of *GhCAD* genes. Motif 1–20 are displayed in different colors.


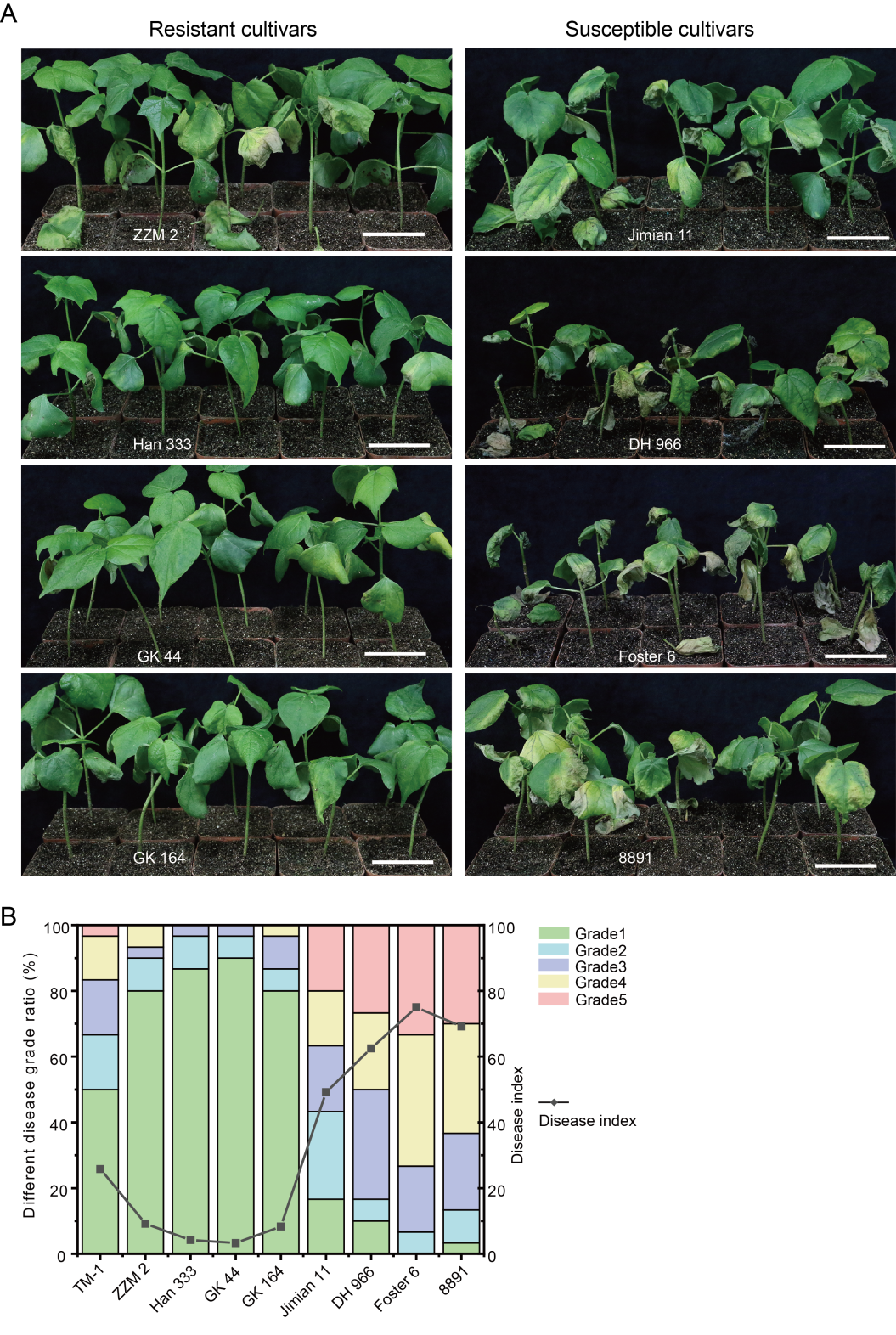


**Figure S2**. Identification of *Verticillium* wilt-resistant and -susceptible cultivars. (A) Phenotypes of *Verticillium* wilt resistant- (left panel) and susceptible-cultivars (right panel) 18 days post-infection (dpi) by *V. dahliae*. (B) Ratio of different diseased grades and disease index for *Verticillium* wilt resistant- and susceptible-cultivars 18 dpi by *V. dahliae*.


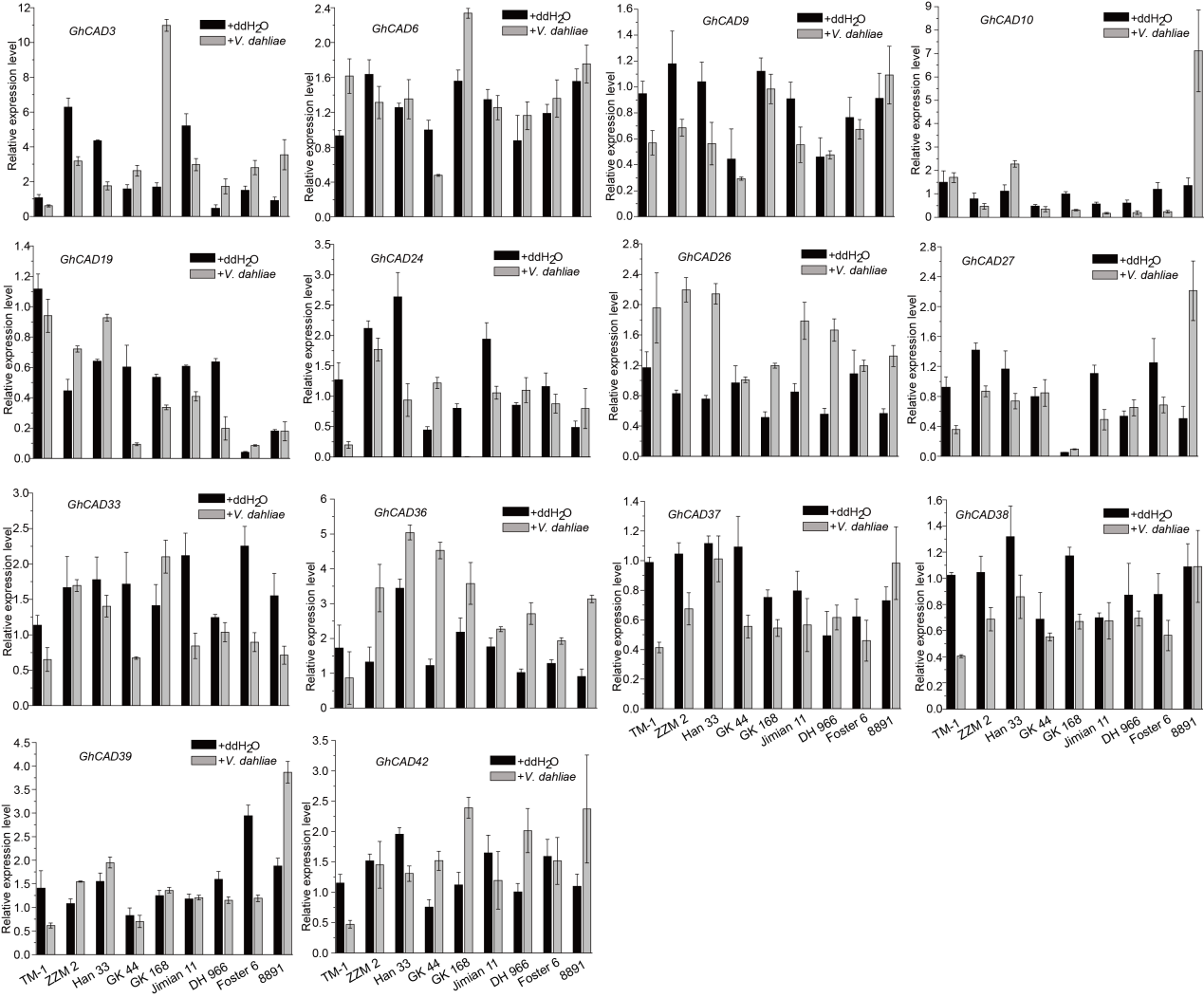


**Figure S3**. Transcriptional levels of different *GhCADs* under the control (ddH_2_O) and *V. dahliae* treatment conditions in different *G. hirsutum* cultivars. Four *Verticillium* wilt resistant cultivars (ZZM 2, Han 333, GK 44, and GK168), four *Verticillium* wilt susceptive cultivars (Jimian 11, DH 966, Foster 6, and 8891) and TM-1 were used in the *V. dahliae* inoculation experiment; two-weeks-old plants 18 days after *V. dahliae* inoculation were used in qRT-PCR analysis. Three biological replicates were performed for each treatment. The gene expression level of each gene in TM-1 under the control (ddH_2_O) treatment was normalized as 1. *GhUBQ7* was used as reference gene.


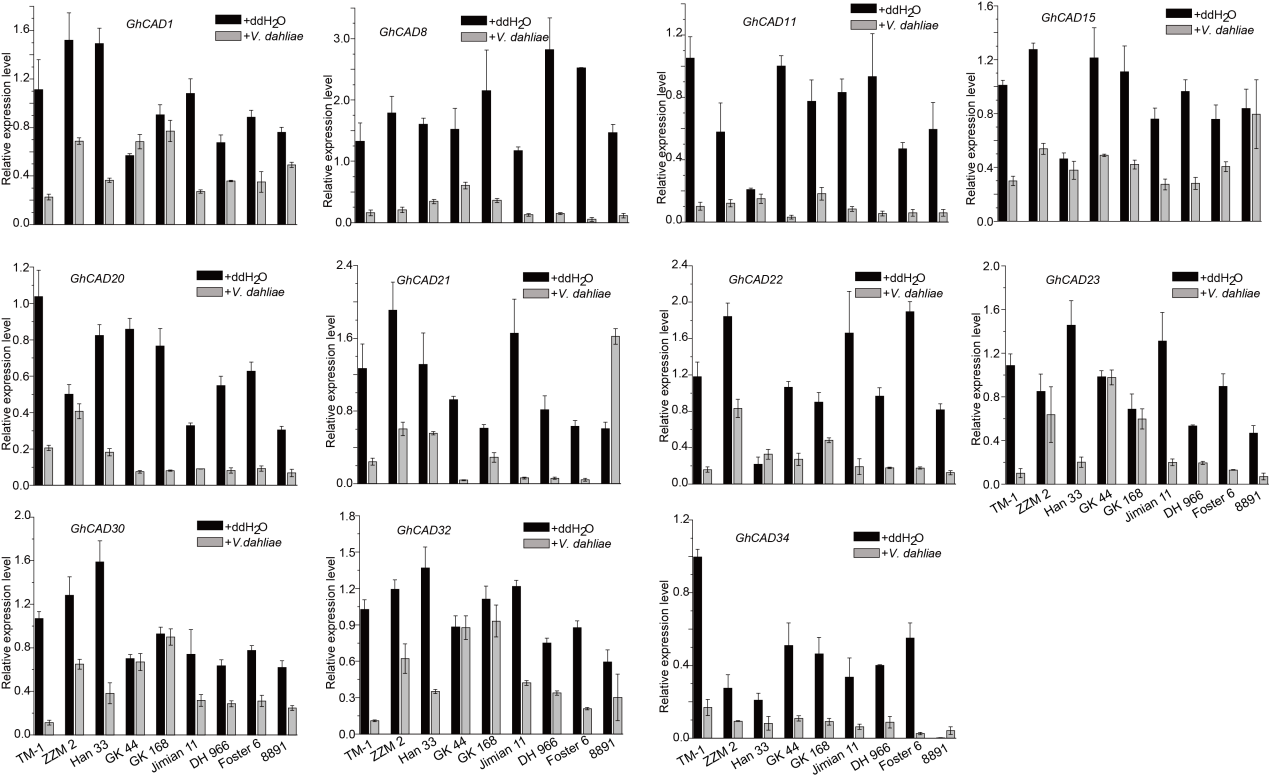


**Figure S4**. Transcriptional levels of different *GhCADs* under the control (ddH_2_O) and *V. dahliae* treatment conditions in different *G. hirsutum* cultivars. Four *Verticillium* wilt resistant cultivars (ZZM 2, Han 333, GK 44, and GK168), four *Verticillium* wilt susceptive cultivars (Jimian 11, DH 966, Foster 6, and 8891) and TM-1 were used in the *V. dahliae* inoculation experiment; two-weeks-old plants 18 days after *V. dahliae* inoculation were used in qRT-PCR analysis. Three biological replicates were performed for each treatment. The gene expression level of each gene in TM-1 under the control (ddH_2_O) treatment was normalized as 1. *GhUBQ7* was used as reference gene.


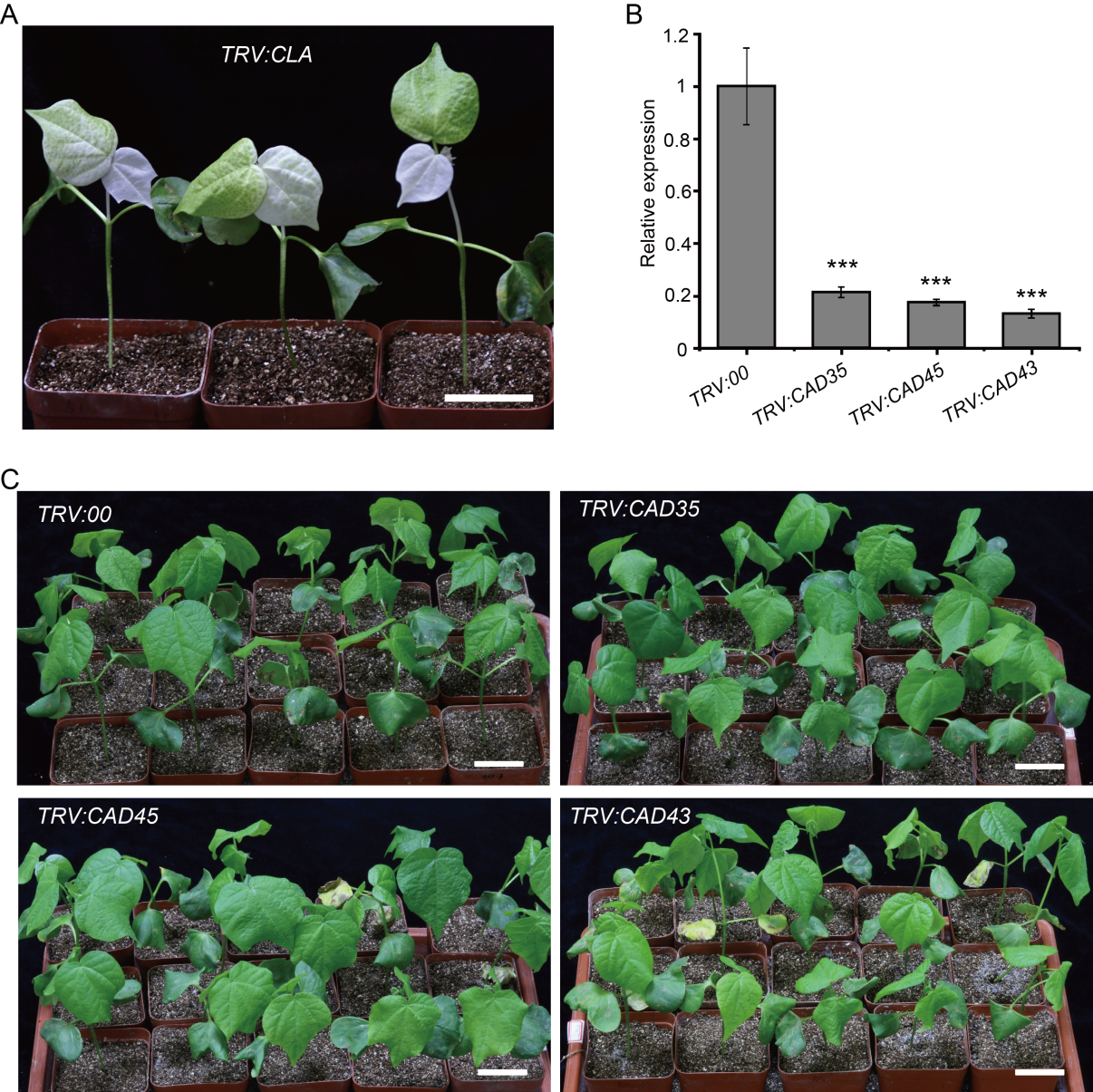


**Figure S5**. Evaluation of the VIGS system. (A) *TRV:CLA* VIGS plants displayed albino phenotypes for the first pair of true leaves. (B) Expression of *GhCAD35*, *GhCAD45* and *GhCAD43* in the corresponding *TRV:CAD35*, *TRV:CAD45* and *TRV:CAD43* VIGS plants relative to those of *TRV:00*, respectively. (C) Phenotypes of the two-weeks-old VIGS-plants. Scale bars, 2 cm. A resistant cultivar Zhongzhimian 2 was used as VIGS background here.


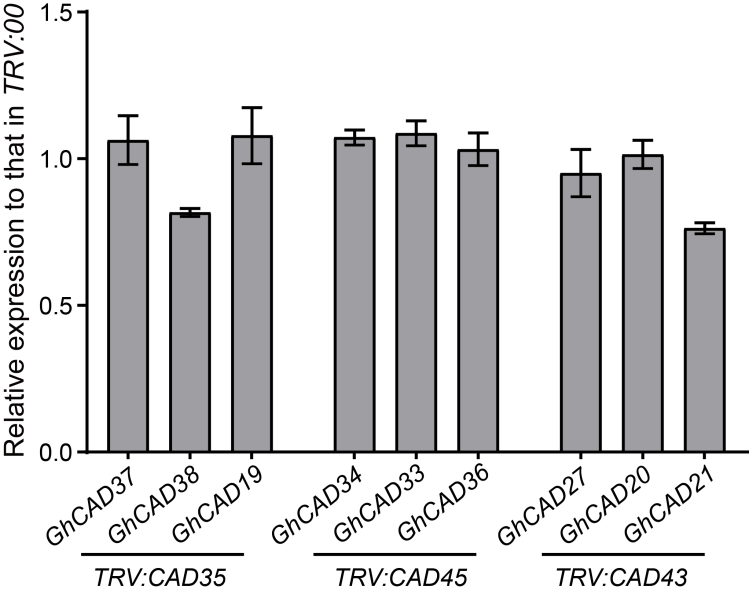


**Figure S6**. The specificity of the gene silencing in the VIGS plants. Expression of three most homologous *GhCAD*s of *GhCAD35*, *GhCAD45*, and *GhCAD43*, respectively, in the corresponding *TRV:CAD35*, *TRV:CAD45* and *TRV:CAD43* VIGS plants relative to those of *TRV:00*.


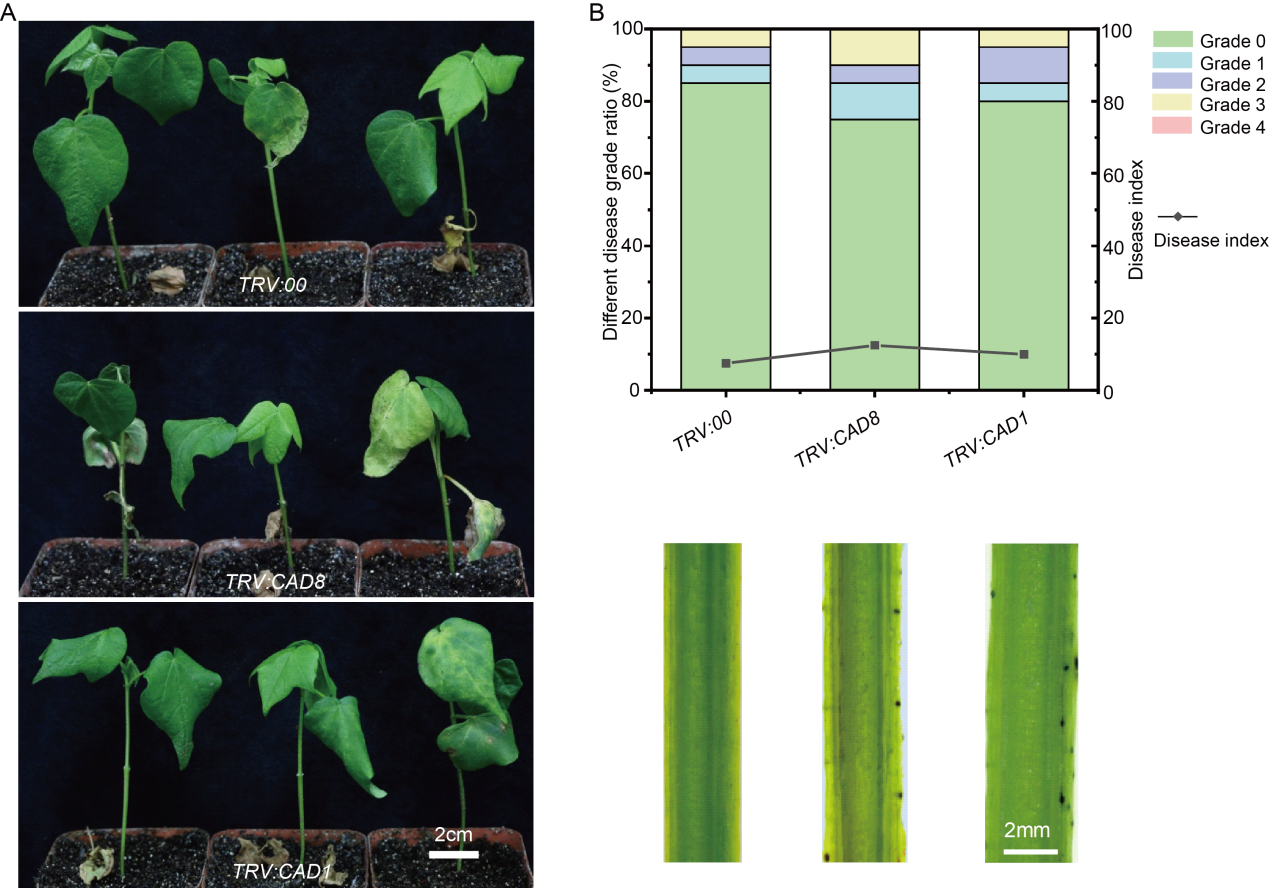


**Figure S7**. *TRV:CAD8* and *TRV:CAD1* plants displayed no different response to *V. dahliae* compared to *TRV:00*. (A) Phenotypes of the *TRV:00*, *TRV:CAD8* and *TRV:CAD1* VIGS plants at 18 dpi by *V. dahliae*. (B) Ratio of different diseased grades and disease index for the *TRV:00*, *TRV:CAD8* and *TRV:CAD1* VIGS plants at 18 dpi by *V. dahliae* (upper panel); the images showing the longitudinal sections of stems of *TRV:00*, *TRV:CAD8* and *TRV:CAD1* VIGS plants 18 dpi by *V. dahliae* (lower panel). A resistant cultivar Zhongzhimian 2 was used as VIGS background here.


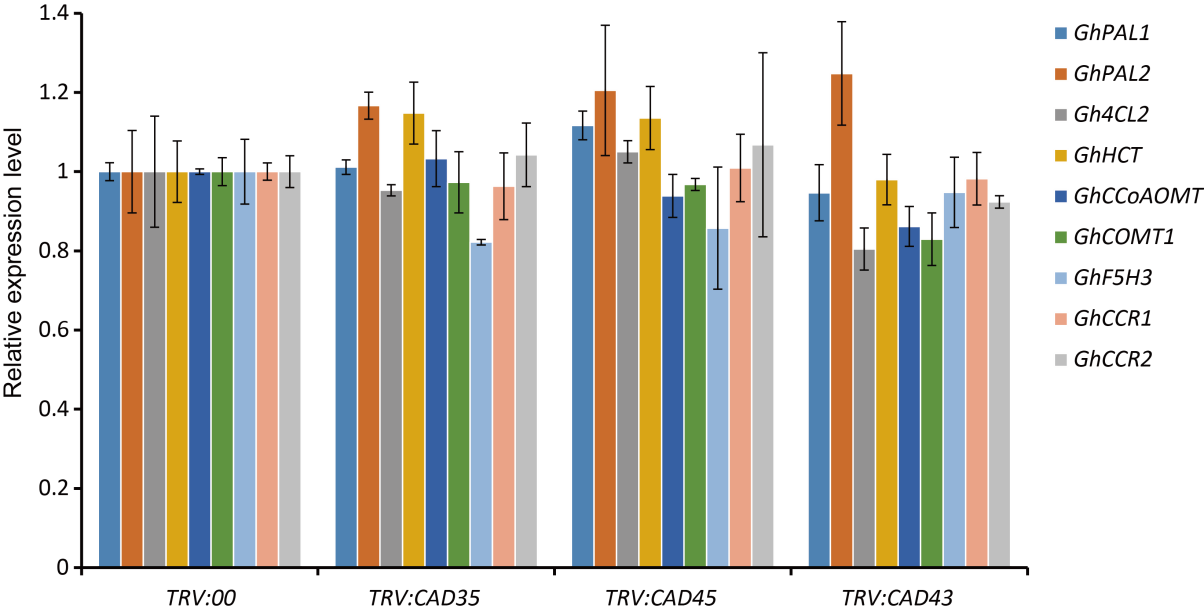


**Figure S8**. The expression of phenylpropanoid metabolic genes in *TRV:00*, *TRV:CAD35*, *TRV:CAD45*, and *TRV:CAD43* VIGS plants.


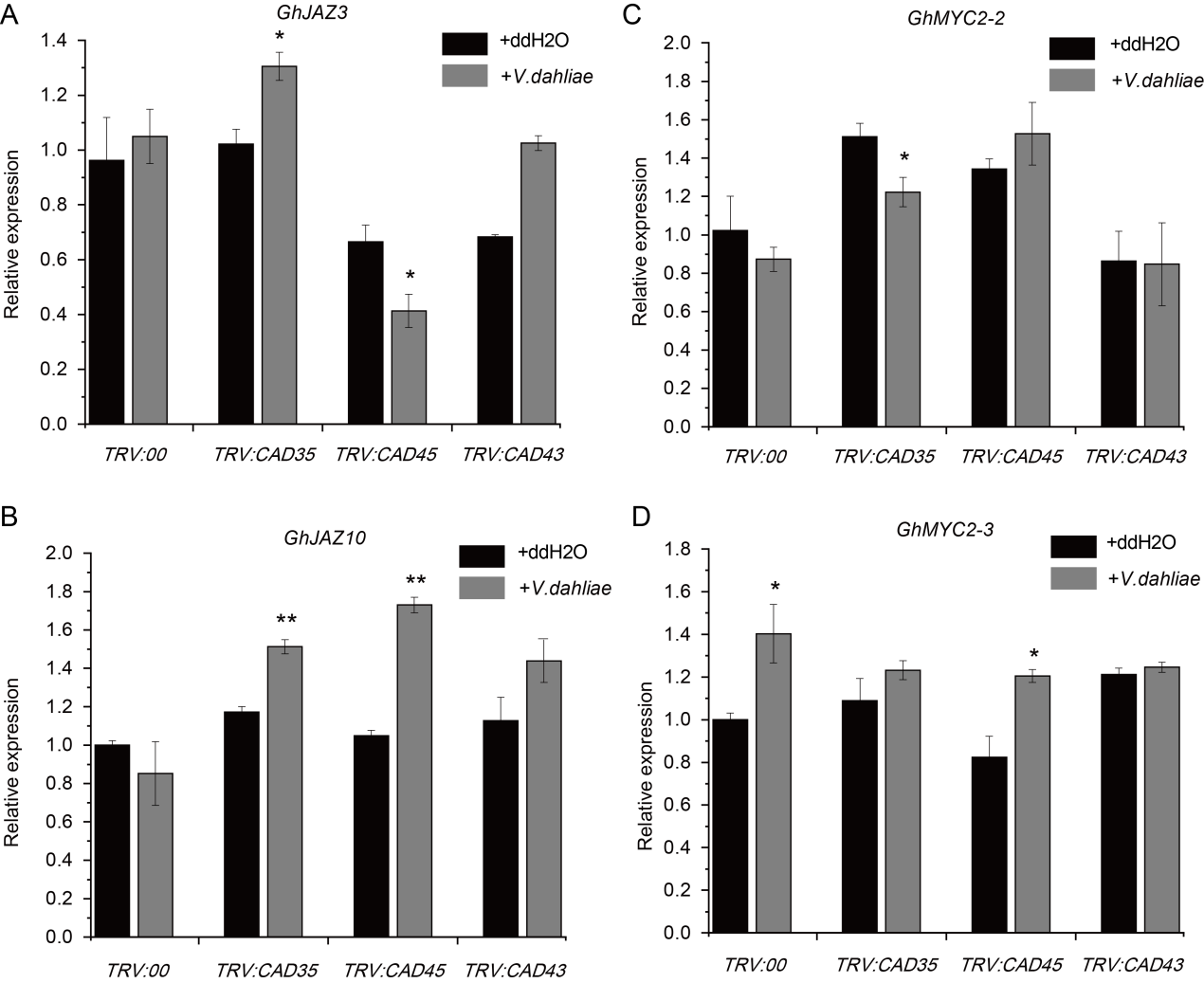


**Figure S9**. The gene expression of JA signaling components *GhJAZ3, GhJAZ10, GhMYB2-2*, and *GhMYB2-3* in *TRV:00*, *TRV:CAD35*, *TRV:CAD45*, and *TRV:CAD43* VIGS plants 12-hours post-infection by *V. dahliae*. The data represent the mean ± SD (n=3 biological replicates), ‘*’ *P* < 0.05, ‘**’ *P* < 0.01 (Student’s *t*-test).


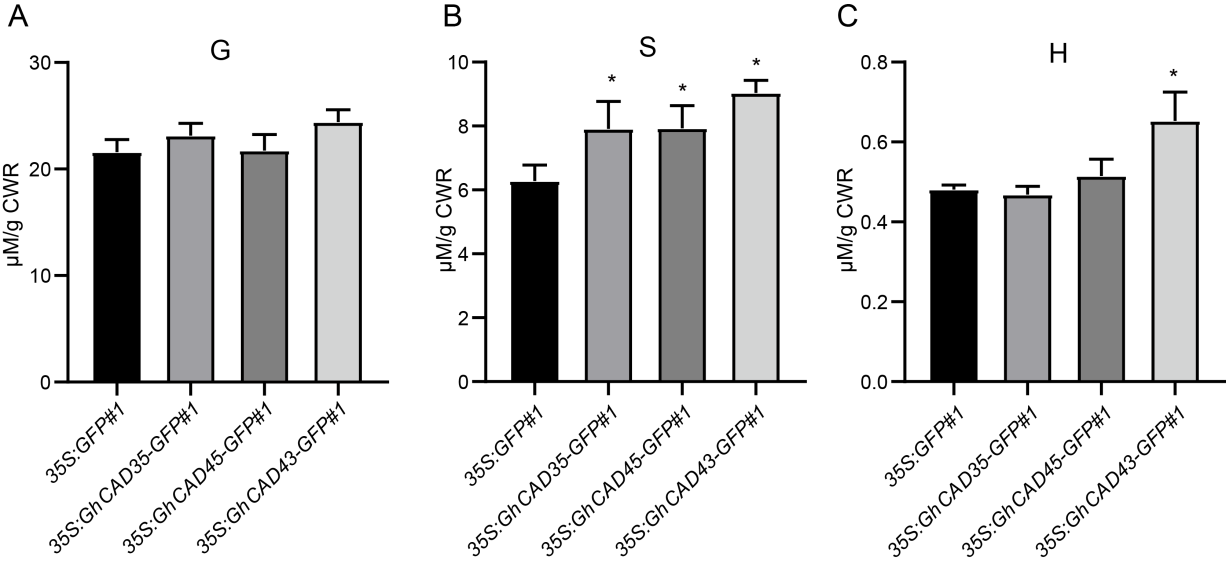


**Figure S10**. Quantitative analyses of H-, G-, and S-units of lignin in the stem tissues from *Arabidopsis* transgenic plants. (A-C) Quantification of H-, G-, and S-units of lignin in the stem tissues *35S:GFP#1*, *35S:GhCAD35-GFP#1*, *35S:GhCAD45-GFP#1*, and *35S:GhCAD43-GFP#1*. Four-week-old Arabidopsis plants grown in long-day photoperiod were used for lignin measurement. The data represent the mean ± SD (n=3), ‘*’ *P* < 0.05 (Student’s *t*-test).
